# Supplementary material for: scDrugPrio: a framework for the analysis of single-cell transcriptomics to address multiple problems in precision medicine in immune-mediated inflammatory diseases
Source: Genome Med. 2024 Mar 20;16:42. doi: 10.1186/s13073-024-01314-7 (PMC10956347; doi:10.1186/s13073-024-01314-7)
Supplement: Supplementary file 1 — Additional file 1: Supplementary figures. Including supplementary figures (Fig. S1-S22) and figure legends. [file 13073_2024_1314_MOESM1_ESM.pdf]

Supplementary Figures

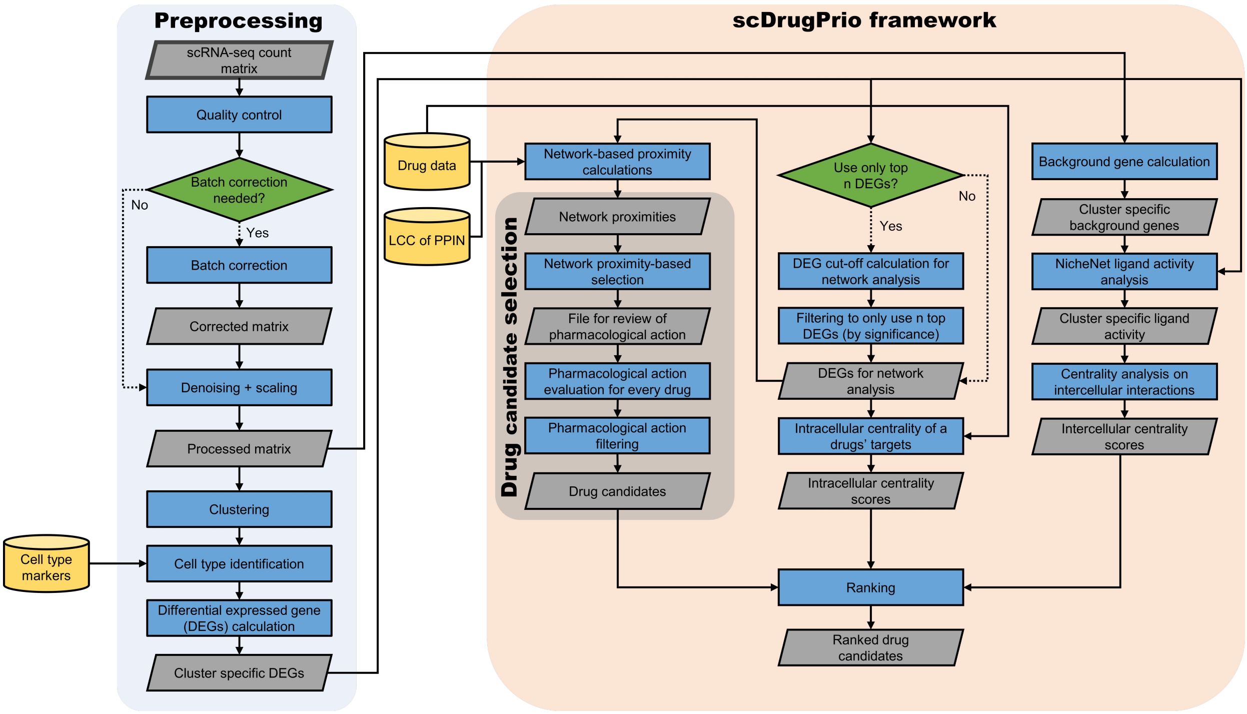

Fig. S1. scDrugPrio computational workflow, input and output data. Initially, data are preprocessed following a standard approach, resulting in the calculation of cell type-specific differentially expressed genes (DEGs) between sick and healthy cells. The need for batch correction was evaluated through heterogeneity analyses as described in **Additional file 2**. The DEG calculation utilized either paired samples (e.g., one healthy and one sick sample) from an individual or pooled samples from several healthy controls and patients. The scDrugPrio framework then performs drug selection using DEGs and drug data for network proximity calculation. The cell type-specific drug candidates are aggregated into a final drug ranking using intracellular and intercellular centrality. Gray parallelograms represent data files, yellow cylinders represent external data and green rombs indicate decision points.

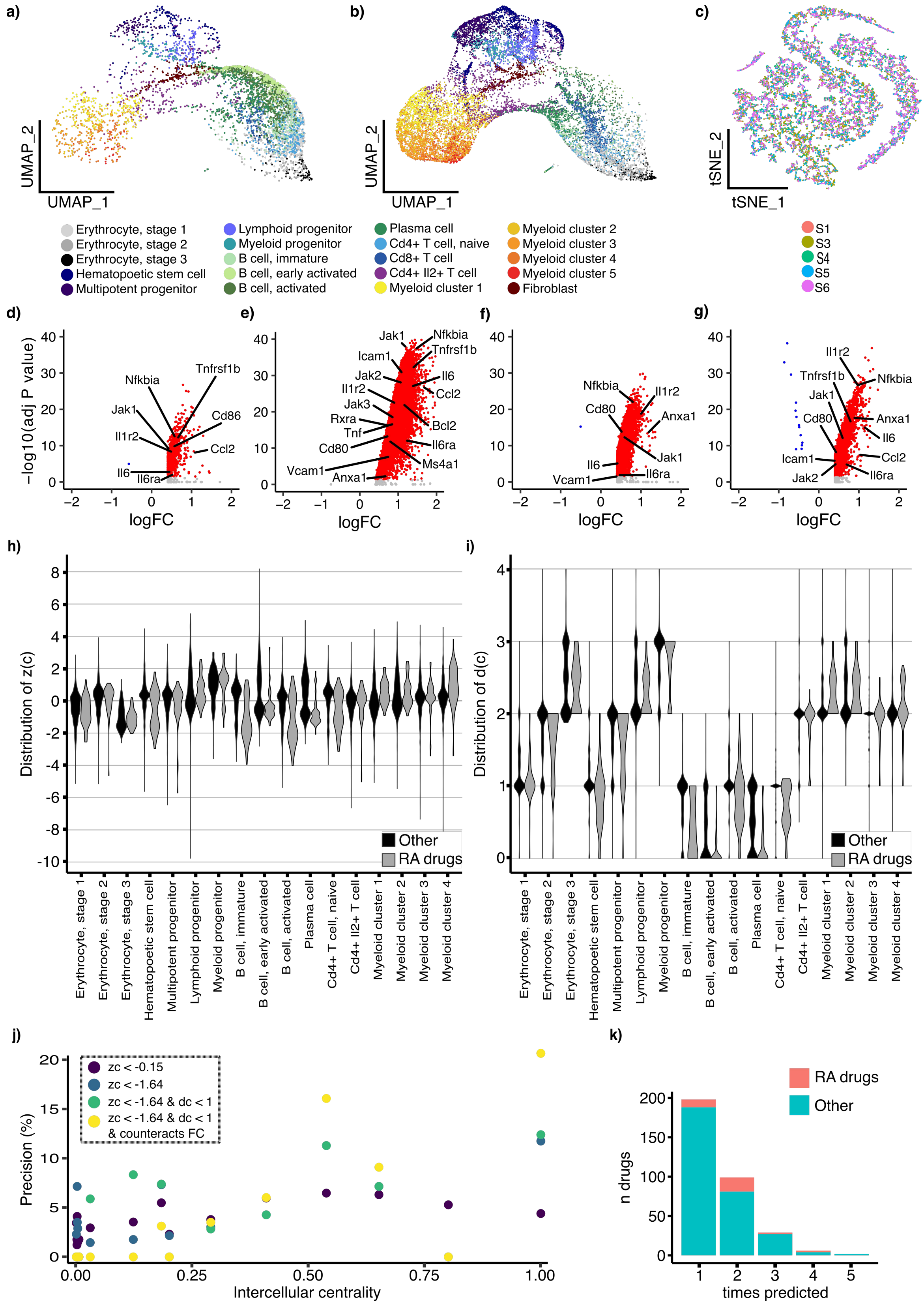

a)
b)
c)
UMAP_2
UMAP_1
tSNE_2
tSNE_1
Erythrocyte, stage 1
Erythrocyte, stage 2
Erythrocyte, stage 3
Hematopoetic stem cell
Multipotent progenitor
Lymphoid progenitor
Myeloid progenitor
B cell, immature
B cell, early activated
B cell, activated
Plasma cell
Cd4+ T cell, naive
Cd8+ T cell
Cd4+ Il2+ T cell
Myeloid cluster 1
Myeloid cluster 2
Myeloid cluster 3
Myeloid cluster 4
Myeloid cluster 5
Fibroblast
S1
S3
S4
S5
S6
d)
e)
f)
g)
−log10(adj P value)
logFC
Tnfrsf1b
Nfkbia
Jak1
Cd86
Il1r2
Ccl2
Il6
Il6ra
Icam1
Jak2
Jak3
Rxra
Tnf
Cd80
Vcam1
Anxa1
Bcl2
Ms4a1
h)
i)
Distribution of z(c)
Distribution of d(c)
Other
RA drugs
j)
k)
zc < -0.15
zc < -1.64
zc < -1.64 & dc < 1
zc < -1.64 & dc < 1
& counteracts FC
Precision (%)
Intercellular centrality
n drugs
times predicted

Fig. S2. Additional cluster information as well as network proximities between rheumatoid arthritis (RA) drugs and DEGs of AIA mice. UMAP plots including only cells from **a**) control mice or **b)** AIA mice. **c)** tSNE representation of cells' latent features of AIA mice to explore interindividual heterogeneity. Cells were colored based on the AIA mouse from which they originated (S1-S6; as indicated by legend). As cells from different AIA mice overlap nicely, no heterogeneity is apparent. **d–g)** Volcano plots of DEGs (FC > 1.5 and FDR-adjusted $P < 0.05$) that are upregulated (red) or downregulated (blue) in B-cell clusters. DEGs that were targeted by approved RA drugs are indicated by arrows. Plots present the DEGs for **d)** activated B cells, **e)** early activated B cells, **f)** plasma cells, and **g)** immature B cells. **h&i)** Violin plots showing z_c and d_c distributions, respectively, for each cell type. **j)** Precision among cell type drug candidates correlated with the cell type eigenvector centrality in the MCDM. Drug candidates were selected using four different sets of criteria, namely, 1) the basic z_c cut-off of $z_c < -0.15$ suggested by Guney et al.(1), 2) $z_c < -1.64$ (corresponding to $P < 0.05$), 3) drugs that fulfil both $z_c < -1.64$ and $d_c < 1$ and 4) drugs fulfilling our final selection criteria ($z_c < -1.64$, $d_c < 1$ and counteracting the fold change (FC) of at least one targeted DEG). Pearson correlation was calculated based on drugs passing the final selection criteria (Pearson's r [95% CI] = 0.77 [0.46 – 0.91], $P < 10^{-3}$). **g)** The proportion of known RA drugs was increased among final candidates that reoccurred in several cell types.

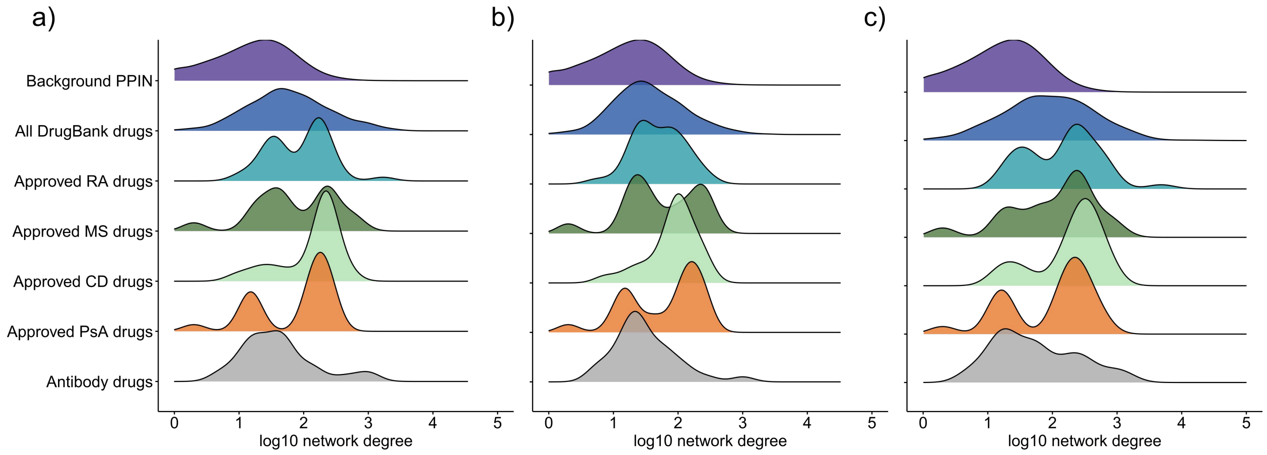

Fig. S3 Distribution of network degrees. In this figure, we visualize the distributions for **a)** max, **b)** mean and **c)** sum of drug targets' network degrees compared to the distribution of network degrees in the LCC of the literature-derived PPIN. As updated treatment regimens for inflammatory diseases often have antibody-based drugs as cornerstones, we visualised their network degrees separately. A total of 316 unique antibody drugs were identified by screening DrugBank for drug names ending with -ab.

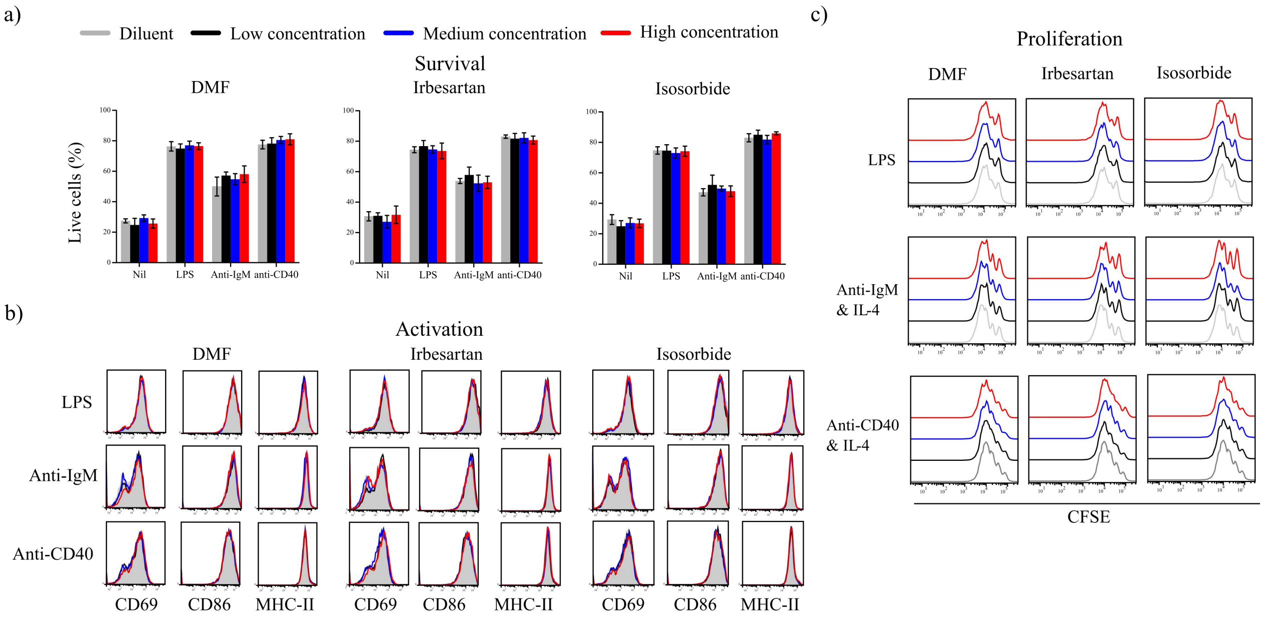

Fig. S4. Dimethyl fumarate (DMF), irbesartan and isosorbide have no effect on murine B-cell viability, activation or proliferation. Drug effect of the selected drugs on *in vitro* murine (**a**) B-cell survival, (**b**) activation, and (**c**) proliferation. Purified murine B cells were stimulated with the indicated B-cell modulators in the presence of dimethyl fumarate (DMF), irbesartan, and isosorbide at different concentrations. Drug concentrations can be found in **Table S2**.

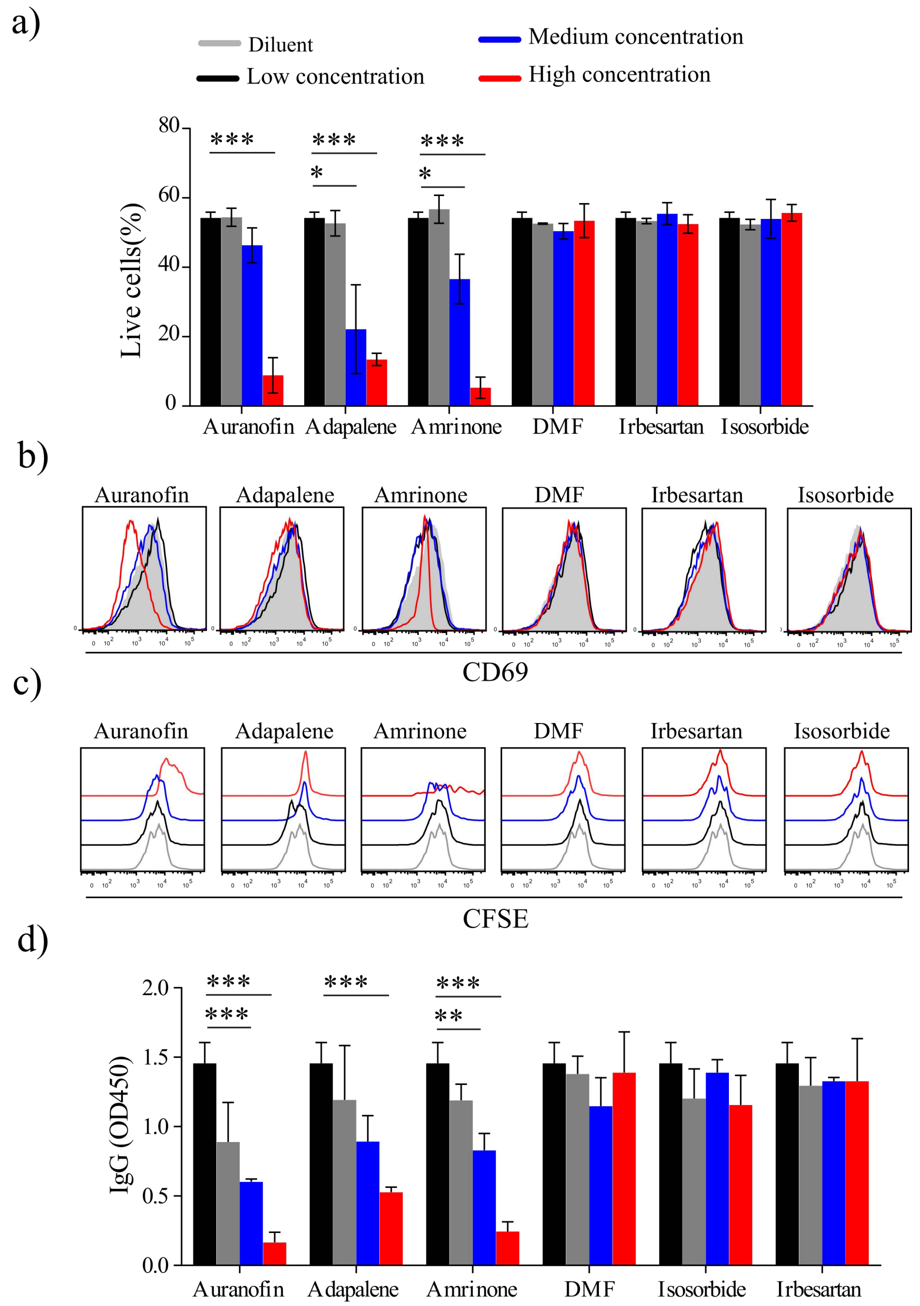

Fig. S5. *In vitro* analysis of selected drugs on human B-cell (a) viability, (b) activation, (c) proliferation and (d) IgG production. Purified human naïve B cells were activated with goat anti-human IgG + IgM (5 μg/mL), anti-human CD40 (5 μg/mL) and IL-21 (10 ng/mL) in the presence of selected drugs at the indicated concentrations. Drug concentrations can be found in **Table S**2. DMF, dimethyl furamate. * $P < 0.05$, **$P < 0.01$, ***$P < 0.001$.

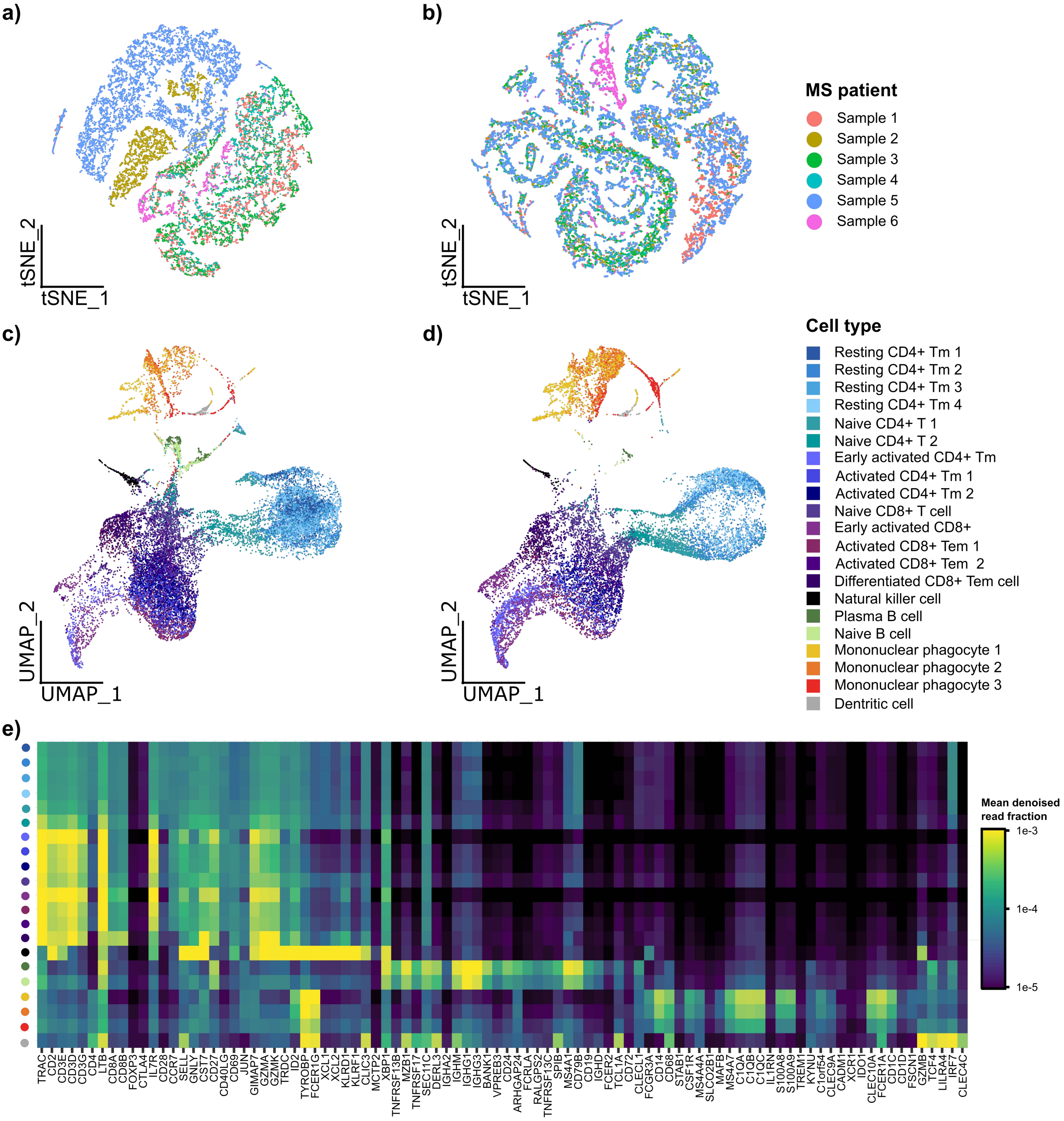
a)
b)
tSNE_2
tSNE_1
tSNE_2
tSNE_1
MS patient
Sample 1
Sample 2
Sample 3
Sample 4
Sample 5
Sample 6
c)
d)
UMAP_2
UMAP_1
UMAP_2
UMAP_1
Cell type
Resting CD4+ Tm 1
Resting CD4+ Tm 2
Resting CD4+ Tm 3
Resting CD4+ Tm 4
Naive CD4+ T 1
Naive CD4+ T 2
Early activated CD4+ Tm
Activated CD4+ Tm 1
Activated CD4+ Tm 2
Naive CD8+ T cell
Early activated CD8+
Activated CD8+ Tem 1
Activated CD8+ Tem 2
Differentiated CD8+ Tem cell
Natural killer cell
Plasma B cell
Naive B cell
Mononuclear phagocyte 1
Mononuclear phagocyte 2
Mononuclear phagocyte 3
Dentritic cell
e)
Mean denoised read fraction
1e-3
1e-4
1e-5
TRAC CD2 CD3E CD3D CD3G CD4 LTB CD8A CD8B FOXP3 CTLA4 IL7R CD28 CCR7 SELL GNLY CST7 CD27 CD40LG CD69 JUN GIMAP7 GZMA GZMK TRDC ID2 TYROBP FCER1G XCL1 XCL2 KLRD1 KLRF1 CLIC3 MCTP2 XBP1 TNFRSF13B MZB1 TNFRSF17 SEC11C DERL3 IGHA2 IGHM IGHG1 IGHG3 BANK1 VPREB3 CD24 ARHGAP24 FCRLA RALGPS2 TNFRSF13C SPIB MS4A1 CD79B CD19 IGHD FCER2 TCL1A CD72 CLECL1 FCGR3A CD14 CD68 STAB1 CSF1R MS4A4A SLCO2B1 MAFB MS4A7 C1QA C1QB C1QC IL1RN S100A8 S100A9 TREM1 KYNU C1orf54 CLEC9A CADM1 XCR1 IDO1 CLEC10A FCER1A CD1C CD1D FSCN1 GZMB TCF4 LILRA4 IRF7 CLEC4C

Fig. S6. Cell typing and selection of the number of DEGs for network-based drug screening in scRNA-seq data from human multiple sclerosis (MS) patients. tSNE representation of latent features for MS patients **a)** before and **b)** after batch correction. Cells were coloured based on which MS patient they originated from (patient 1 to 6). In contrast to **Fig. S1c,** we can see that cells from different patients do not mix well before batch correction (**a**), indicating transcriptomic heterogeneity between patients. UMAP clustering plots corresponding to **Fig. 5a** including only cerebrospinal fluid cells from **c**) MS patients or **d)** controls (idiopathic intracranial hypertension patients). **e)** Cell typing by cluster means of DCA-denoised, log10 adjusted gene expression fractions for known marker genes. Abbreviations: Tm, memory T cell; Tem, effector memory T cell.

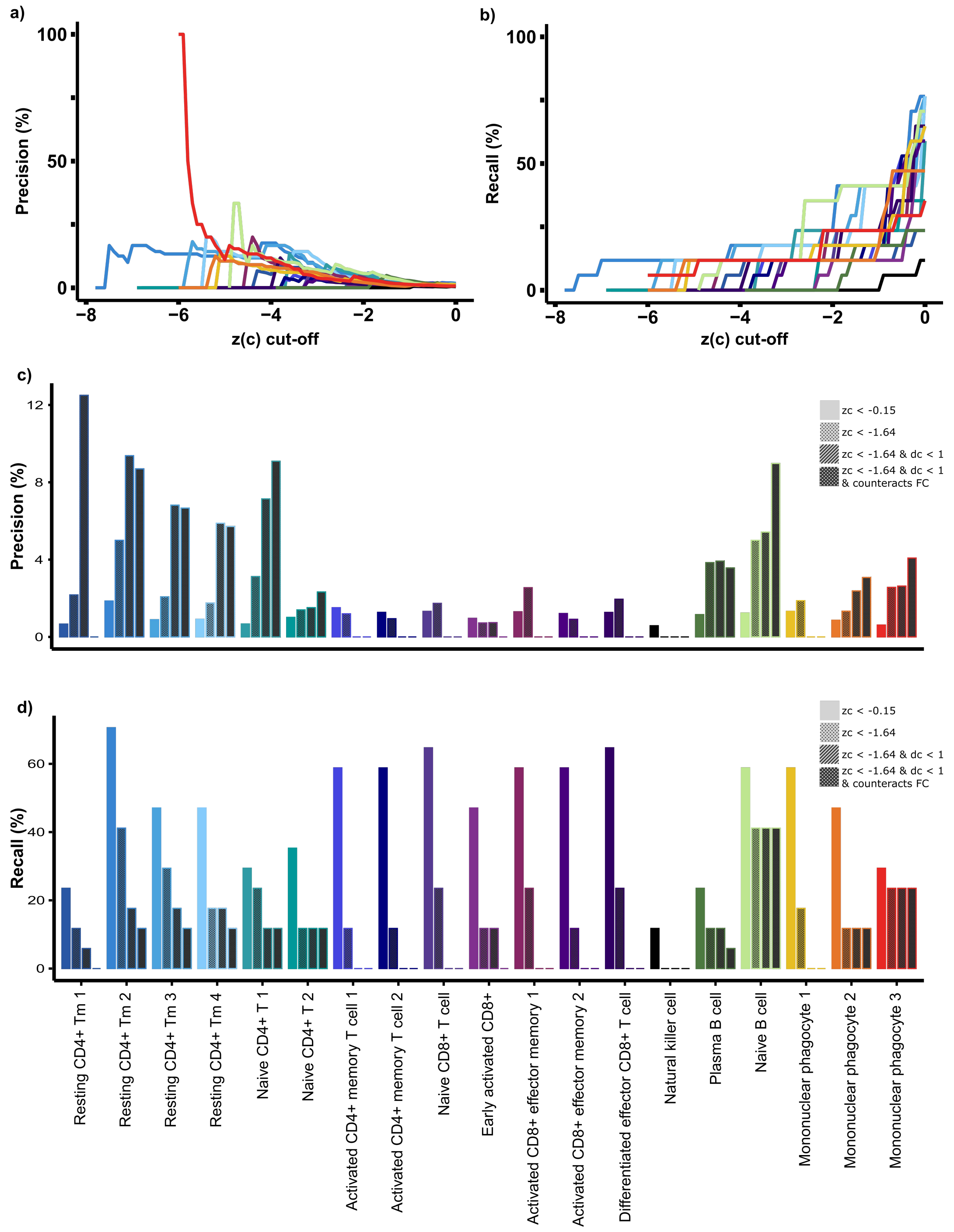

Fig. S7. Drug candidate selection for multiple sclerosis (MS) patients. a) Precision and **b)** recall curves for approved drugs among candidates at different z_c cut-offs indicate that decreasing z_c increases precision among central cell types in the MCDM. **c&d)** Precision (c) and recall (d) after stepwise application of drug selection criteria to candidates selected based on network distances to the top 3,000 significant DEGs.

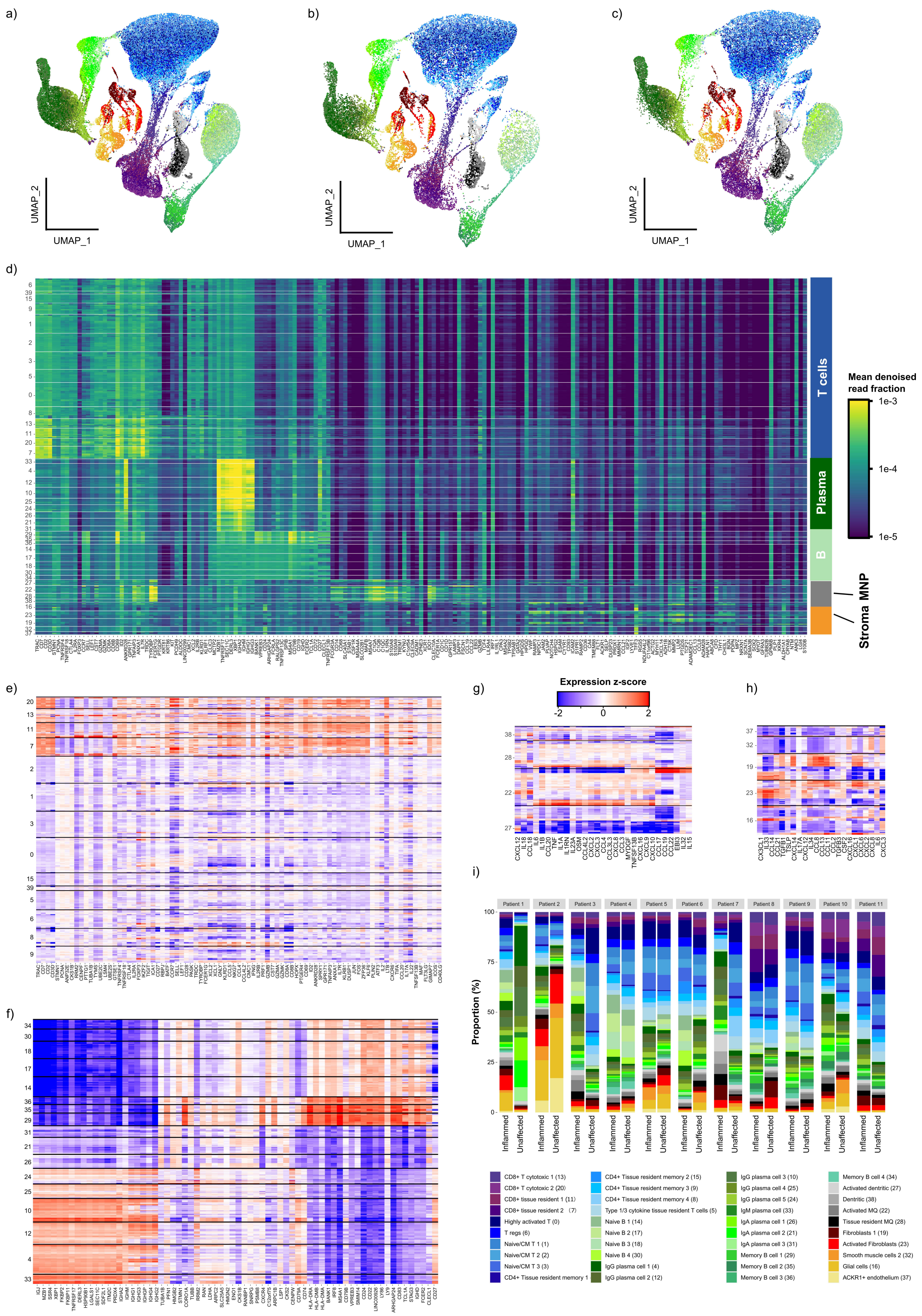

a)
b)
c)
UMAP_2
UMAP_1
d)
T cells
Plasma
B
Stroma MNP
Mean denoised read fraction
1e-3
1e-4
1e-5
e)
f)
g)
h)
Expression z-score
-2
0
2
i)
Patient 1
Patient 2
Patient 3
Patient 4
Patient 5
Patient 6
Patient 7
Patient 8
Patient 9
Patient 10
Patient 11
Proportion (%)
100
75
50
25
0
Inflammed
Unaffected
CD8+ T cytotoxic 1 (13)
CD8+ T cytotoxic 2 (20)
CD8+ tissue resident 1 (11)
CD8+ tissue resident 2 (7)
Highly activated T (0)
T regs (6)
Naive/CM T 1 (1)
Naive/CM T 2 (2)
Naive/CM T 3 (3)
CD4+ Tissue resident memory 1
CD4+ Tissue resident memory 2 (15)
CD4+ Tissue resident memory 3 (9)
CD4+ Tissue resident memory 4 (8)
Type 1/3 cytokine tissue resident T cells (5)
Naive B 1 (14)
Naive B 2 (17)
Naive B 3 (18)
Naive B 4 (30)
IgG plasma cell 1 (4)
IgG plasma cell 2 (12)
IgG plasma cell 3 (10)
IgG plasma cell 4 (25)
IgG plasma cell 5 (24)
IgM plasma cell (33)
IgA plasma cell 1 (26)
IgA plasma cell 2 (21)
IgA plasma cell 3 (31)
Memory B cell 1 (29)
Memory B cell 2 (35)
Memory B cell 3 (36)
Memory B cell 4 (34)
Activated dentritic (27)
Dentritic (38)
Activated MQ (22)
Tissue resident MQ (28)
Fibroblasts 1 (19)
Activated Fibroblasts (23)
Smooth muscle cells 2 (32)
Glial cells (16)
ACKR1+ endothelium (37)

Fig. S8. Clustering and cell typing of Crohn's disease patient data. a) tSNE plot including all cells from inflamed and unaffected intestinal lesions of all patients. Clusters are colour coded by cell types in the colour legend of i). **b&c)** are tSNE plots that include only cells from unaffected and inflamed intestinal biopsies. **d)** Heatmap representing individual cell expression of major cell type marker genes. Gene expression values correspond to DCA(2)-adjusted gene expression value fractions of a cell's total DCA-adjusted gene expression values. Individual cells are grouped by the cluster that is represented by a number on the y-axis corresponding to the number in the colour legend of i). White lines separate clusters for visibility. Bars on the right side indicate the major cell type. **e-g)** Heatmaps of single-cell gene expression fraction-based z-score for further stratification of T cells (e), B cells (f), MNPs (g) and stroma/glia cells (h). **i)** Stacked bar plots representing the fractions of cells per cell type and sample. Abbreviations: ILC, innate lymphocyte cells; MNP, mononuclear phagocytes. Heatmap x labels can be found in **Additional file 5**.

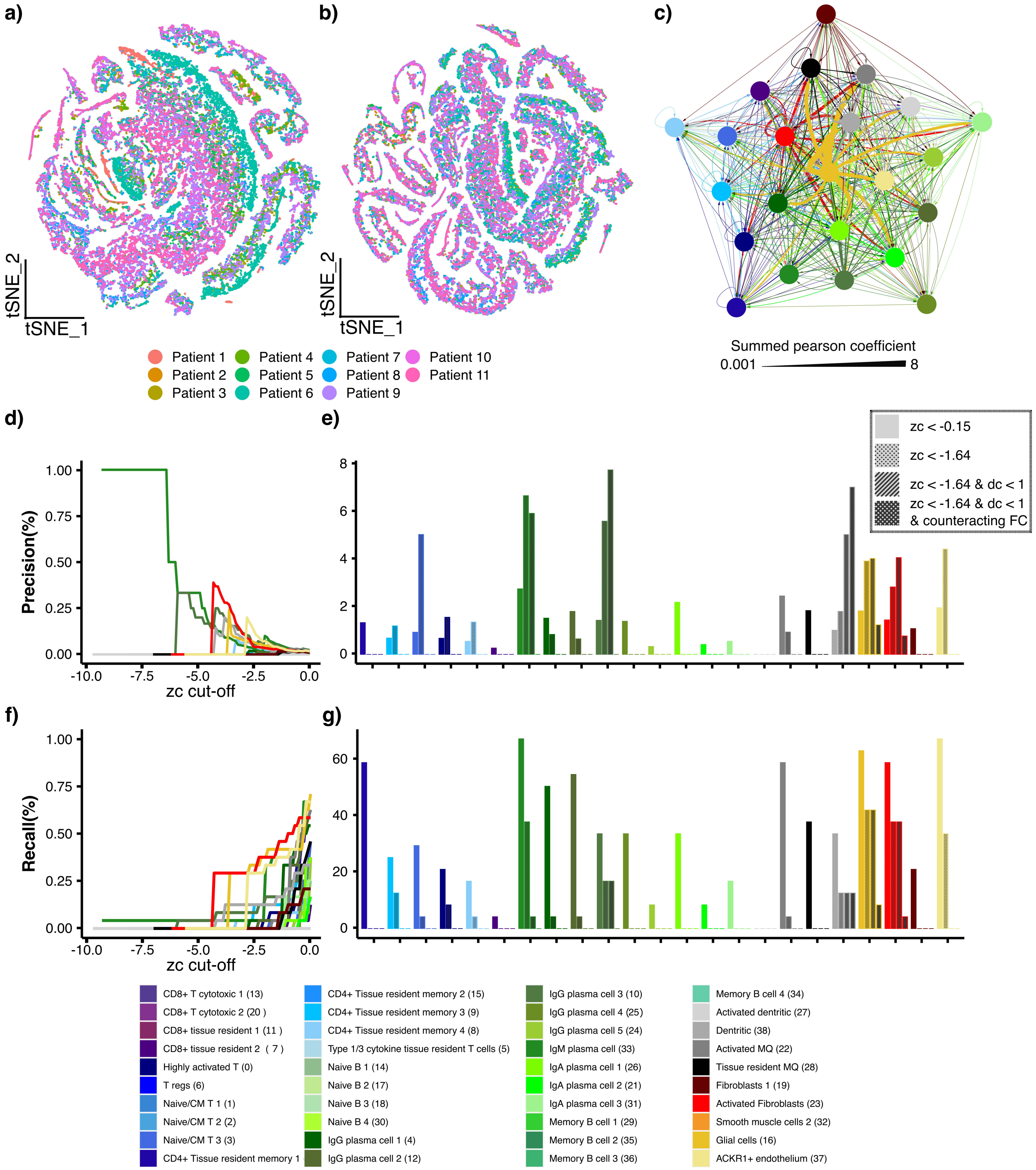
a)
b)
c)
tSNE_2
tSNE_1
tSNE_2
tSNE_1
Patient 1
Patient 2
Patient 3
Patient 4
Patient 5
Patient 6
Patient 7
Patient 8
Patient 9
Patient 10
Patient 11
Summed pearson coefficient
0.001
8
d)
e)
zc < -0.15
zc < -1.64
zc < -1.64 & dc < 1
zc < -1.64 & dc < 1 & counteracting FC
Precision(%)
1.00
0.75
0.50
0.25
0.00
-10.0
-7.5
-5.0
-2.5
0.0
zc cut-off
8
6
4
2
0
f)
g)
Recall(%)
1.00
0.75
0.50
0.25
0.00
-10.0
-7.5
-5.0
-2.5
0.0
zc cut-off
60
40
20
0
CD8+ T cytotoxic 1 (13)
CD8+ T cytotoxic 2 (20)
CD8+ tissue resident 1 (11)
CD8+ tissue resident 2 (7)
Highly activated T (0)
T regs (6)
Naive/CM T 1 (1)
Naive/CM T 2 (2)
Naive/CM T 3 (3)
CD4+ Tissue resident memory 1
CD4+ Tissue resident memory 2 (15)
CD4+ Tissue resident memory 3 (9)
CD4+ Tissue resident memory 4 (8)
Type 1/3 cytokine tissue resident T cells (5)
Naive B 1 (14)
Naive B 2 (17)
Naive B 3 (18)
Naive B 4 (30)
IgG plasma cell 1 (4)
IgG plasma cell 2 (12)
IgG plasma cell 3 (10)
IgG plasma cell 4 (25)
IgG plasma cell 5 (24)
IgM plasma cell (33)
IgA plasma cell 1 (26)
IgA plasma cell 2 (21)
IgA plasma cell 3 (31)
Memory B cell 1 (29)
Memory B cell 2 (35)
Memory B cell 3 (36)
Memory B cell 4 (34)
Activated dentritic (27)
Dentritic (38)
Activated MQ (22)
Tissue resident MQ (28)
Fibroblasts 1 (19)
Activated Fibroblasts (23)
Smooth muscle cells 2 (32)
Glial cells (16)
ACKR1+ endothelium (37)

Fig. S9. Batch correction and drug candidate selection for Crohn's disease patient data. tSNE representation of cell latent features for CD patients **a)** before and **b)** after batch correction. Cells were coloured based on which CD patient they originated from (patient 1 to 11, see **Additional file 5** for more information). Before batch correction, (**a**) cells from patients do not mix well, indicating underlying differences in gene expression profiles. **c)** MCDM based on NicheNet (3)-derived cellular interactions among cell types. Nodes represent cell types, and directed edges indicate ligand interactions from NicheNet. Edge width corresponds to the summed Pearson correlation coefficient of all ligands in the upstream cell type having a potential effect on the downstream cell type. **d&f)** Precision & recall curves at different z_c cut-offs when entering only the top 1,800 DEGs into network distance calculations. **e&g)** Precision and recall for known CD drugs depending on selection criteria (indicated by pattern) using only the top 1,800 significant DEGs for network calculations. Colours in **c-g)** match the colour legend in the bottom.

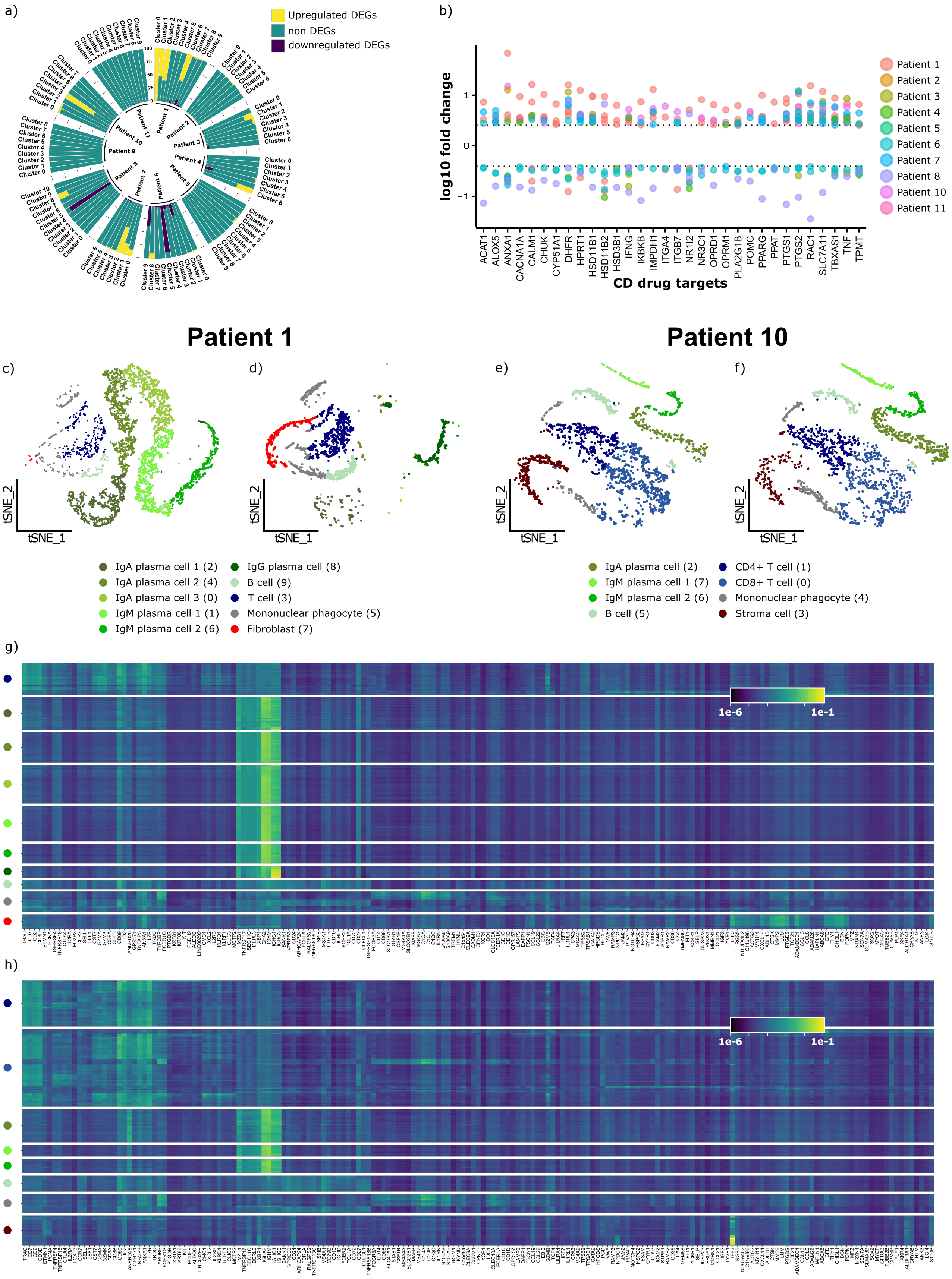

a)
Upregulated DEGs
non DEGs
downregulated DEGs
Patient 1
Patient 2
Patient 3
Patient 4
Patient 5
Patient 6
Patient 7
Patient 8
Patient 9
Patient 10
Patient 11
b)
log10 fold change
CD drug targets
ACAT1
ALOX5
ANXA1
CACNA1A
CALM1
CHUK
CYP51A1
DHFR
HPRT1
HSD11B1
HSD11B2
HSD3B1
IFNG
IKBKB
IMPDH1
ITGA4
ITGB7
NR1I2
NR3C1
OPRD1
OPRM1
PLA2G1B
POMC
PPARG
PPAT
PTGS1
PTGS2
RAC1
SLC7A11
TBXAS1
TNF
TPMT
Patient 1
Patient 2
Patient 3
Patient 4
Patient 5
Patient 6
Patient 7
Patient 8
Patient 10
Patient 11
Patient 1
Patient 10
c)
d)
e)
f)
tSNE_1
tSNE_2
IgA plasma cell 1 (2)
IgA plasma cell 2 (4)
IgA plasma cell 3 (0)
IgM plasma cell 1 (1)
IgM plasma cell 2 (6)
IgG plasma cell (8)
B cell (9)
T cell (3)
Mononuclear phagocyte (5)
Fibroblast (7)
IgA plasma cell (2)
IgM plasma cell 1 (7)
IgM plasma cell 2 (6)
B cell (5)
CD4+ T cell (1)
CD8+ T cell (0)
Mononuclear phagocyte (4)
Stroma cell (3)
g)
1e-6
1e-1
h)
1e-6
1e-1

Fig. S10. Clustering and cell typing of individual Crohn's disease patients. a) Fraction of differentially expressed genes out of all genes. For this, data were divided by patient and clustered separately. For patient 1 and 10, clusters were cell typed, and cluster ID in a) corresponds to the number in parentheses in the figure legend of c-d) and e-f), respectively. **b)** Fold change of known CD drug targets for all patient-specific DEGs in all clusters. The colour represents the patients; in some instances, several clusters of a patient differentially expressed a certain drug target. **c-d)** tSNE plot showing only cells from the uninflamed (c) and inflamed (d) biopsy of patient 1. **e-f)** Same as c-d) for patient 10. **g)** Heatmap representing individual cell expression of major cell type marker genes used for cell typing the clusters of patient 1. Gene expression values correspond to DCA(2) adjusted gene expression value fractions of a cell's total DCA adjusted gene expression values. Individual cells are grouped by cluster. Colours on the y-axis correspond to the colour legend of c-d). Comparable to the heatmap in **Fig. S4d**. **h)** Same as g) for patient 10; colours on the Y-axis correspond to colour legend of e-f).

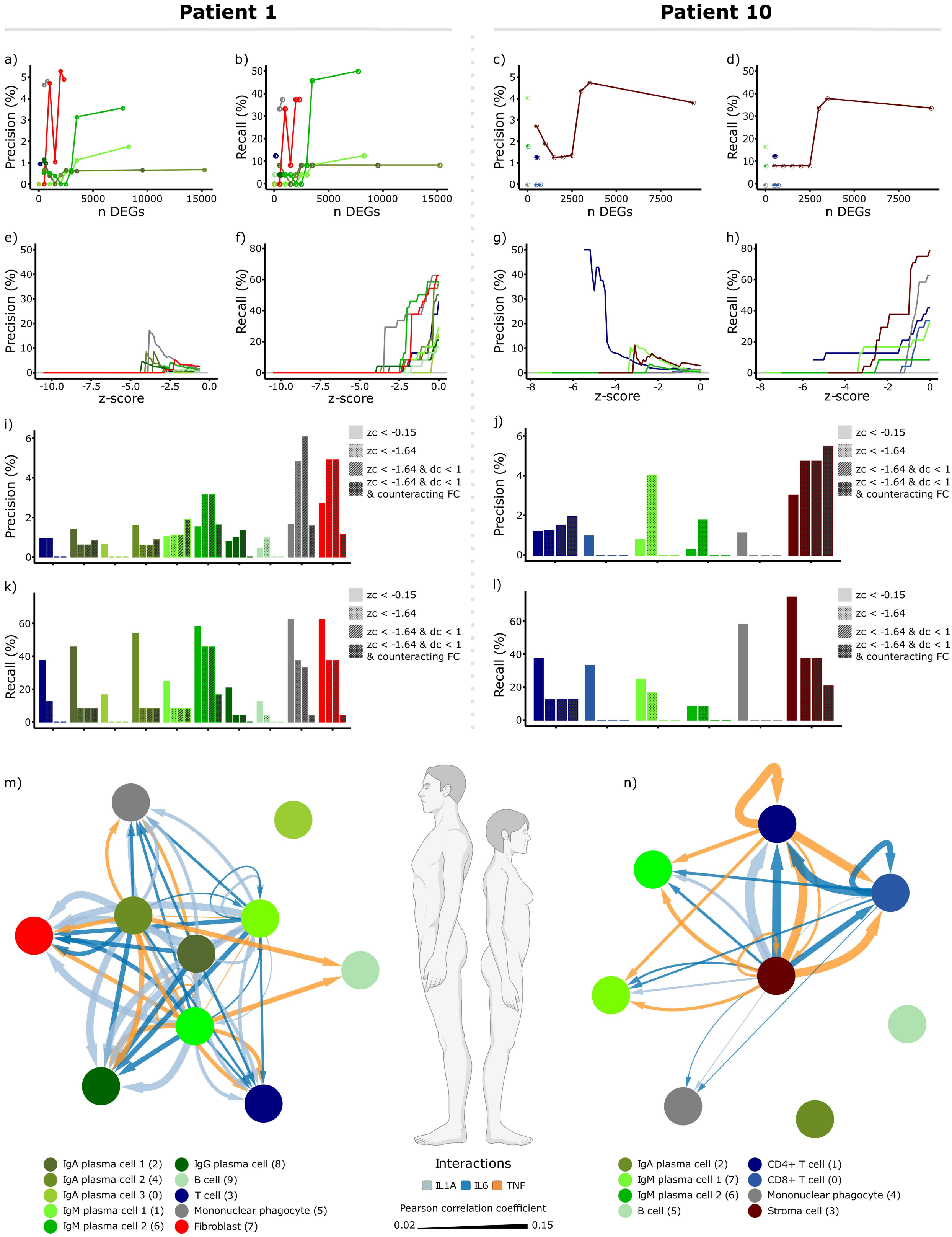

Patient 1
Patient 10
a)
b)
c)
d)
Precision (%)
Recall (%)
n DEGs
e)
f)
g)
h)
z-score
i)
j)
k)
l)
zc < -0.15
zc < -1.64
zc < -1.64 & dc < 1
zc < -1.64 & dc < 1
& counteracting FC
m)
n)
IgA plasma cell 1 (2)
IgA plasma cell 2 (4)
IgA plasma cell 3 (0)
IgM plasma cell 1 (1)
IgM plasma cell 2 (6)
IgG plasma cell (8)
B cell (9)
T cell (3)
Mononuclear phagocyte (5)
Fibroblast (7)
Interactions
IL1A
IL6
TNF
Pearson correlation coefficient
0.02
0.15
IgA plasma cell (2)
IgM plasma cell 1 (7)
IgM plasma cell 2 (6)
B cell (5)
CD4+ T cell (1)
CD8+ T cell (0)
Mononuclear phagocyte (4)
Stroma cell (3)

Fig. S11. **Drug prediction outcomes based on individual Crohn's disease patients**. The panel is divided in the middle and represents outcomes for patients 1 and 10 on the left and right sides, respectively. **a-d)** Cell type precision and recall for known CD drugs at $z_c < -1.64$, shown as a function of the number of an individual patient's top significant DEGs that entered network distance calculations. **e-h)** Precision & recall curves at different z_c cut-offs when entering only the top 3,500 DEGs for each patient into network distance calculations. **i-l)** Precision and recall for known CD drugs depending on selection criteria (indicated by pattern), using only the top 3,500 significant DEGs of each patient for network calculations. **m-n)** MCDMs highlighting only *IL1A*, *IL6* and *TNF* ligand interactions between cell types from patients 1 and 10. Edge width is scaled by the NicheNet (3) Pearson correlation coefficient, which is a measure of each single ligand association with the observed differential gene expression in the downstream cell type.

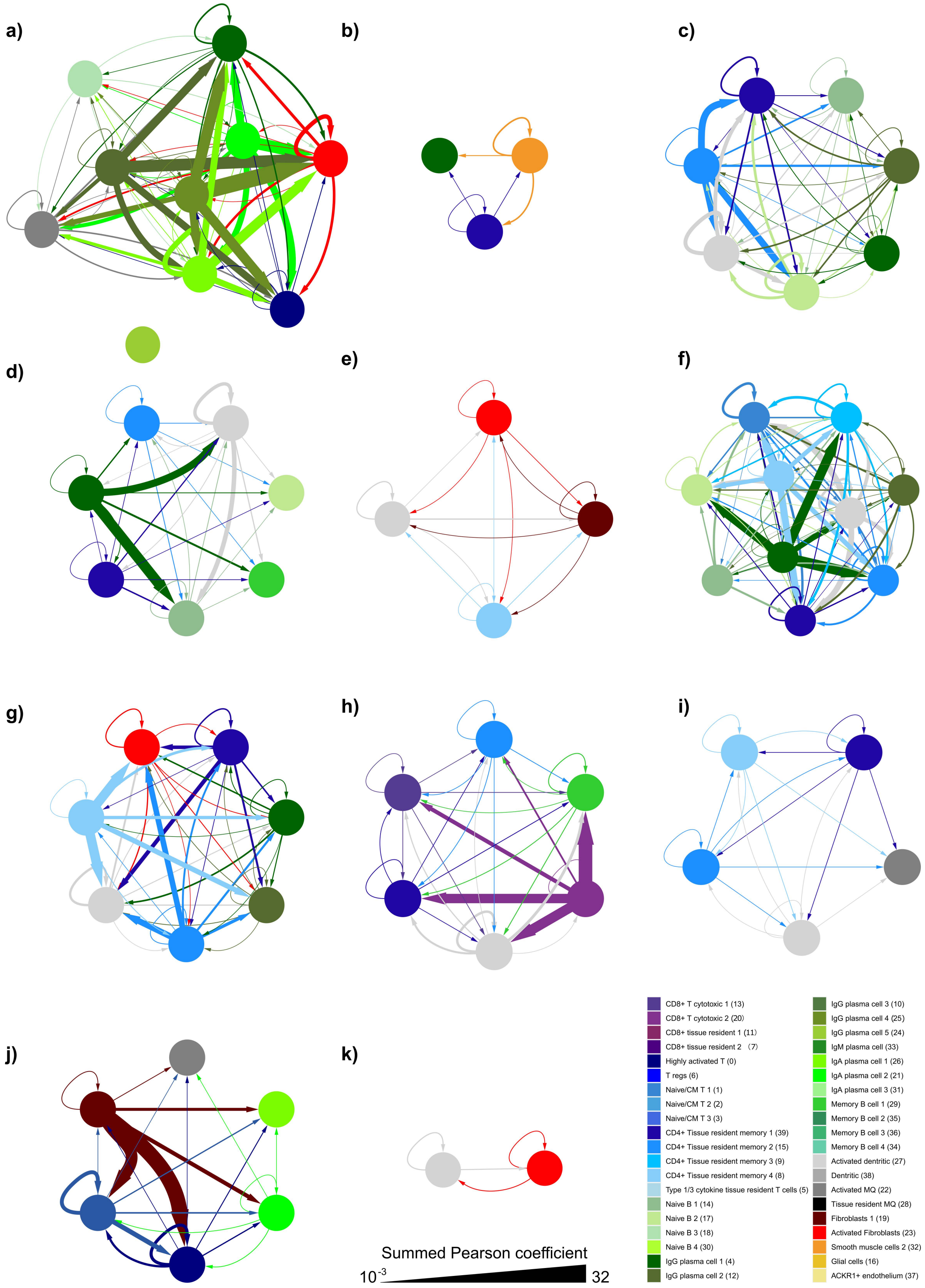
a)
b)
c)
d)
e)
f)
g)
h)
i)
j)
k)
Summed Pearson coefficient
10^{-3}
32
CD8+ T cytotoxic 1 (13)
CD8+ T cytotoxic 2 (20)
CD8+ tissue resident 1 (11)
CD8+ tissue resident 2 (7)
Highly activated T (0)
T regs (6)
Naive/CM T 1 (1)
Naive/CM T 2 (2)
Naive/CM T 3 (3)
CD4+ Tissue resident memory 1 (39)
CD4+ Tissue resident memory 2 (15)
CD4+ Tissue resident memory 3 (9)
CD4+ Tissue resident memory 4 (8)
Type 1/3 cytokine tissue resident T cells (5)
Naive B 1 (14)
Naive B 2 (17)
Naive B 3 (18)
Naive B 4 (30)
IgG plasma cell 1 (4)
IgG plasma cell 2 (12)
IgG plasma cell 3 (10)
IgG plasma cell 4 (25)
IgG plasma cell 5 (24)
IgM plasma cell (33)
IgA plasma cell 1 (26)
IgA plasma cell 2 (21)
IgA plasma cell 3 (31)
Memory B cell 1 (29)
Memory B cell 2 (35)
Memory B cell 3 (36)
Memory B cell 4 (34)
Activated dentritic (27)
Dentritic (38)
Activated MQ (22)
Tissue resident MQ (28)
Fibroblasts 1 (19)
Activated Fibroblasts (23)
Smooth muscle cells 2 (32)
Glial cells (16)
ACKR1+ endothelium (37)

Fig. S12. MCDMs of all individual CD patients. a-k) Correspond to patients 1 to 11. Nodes correspond to cell types that expressed DEGs and are coloured according to the colour legend in the lower right corner. Patient 1 had one cell type that was not connected to the rest of the remaining MCDM. Except for patients 1 and 10, which were cell typed as described in **Fig. S8g&h, c**ell types were derived from the overall cell typing for the pooled patient data (**Fig. S6d**). The directed edges are coloured by colour of the upstream cell type. Edge width corresponds to the summed Pearson correlation coefficient.

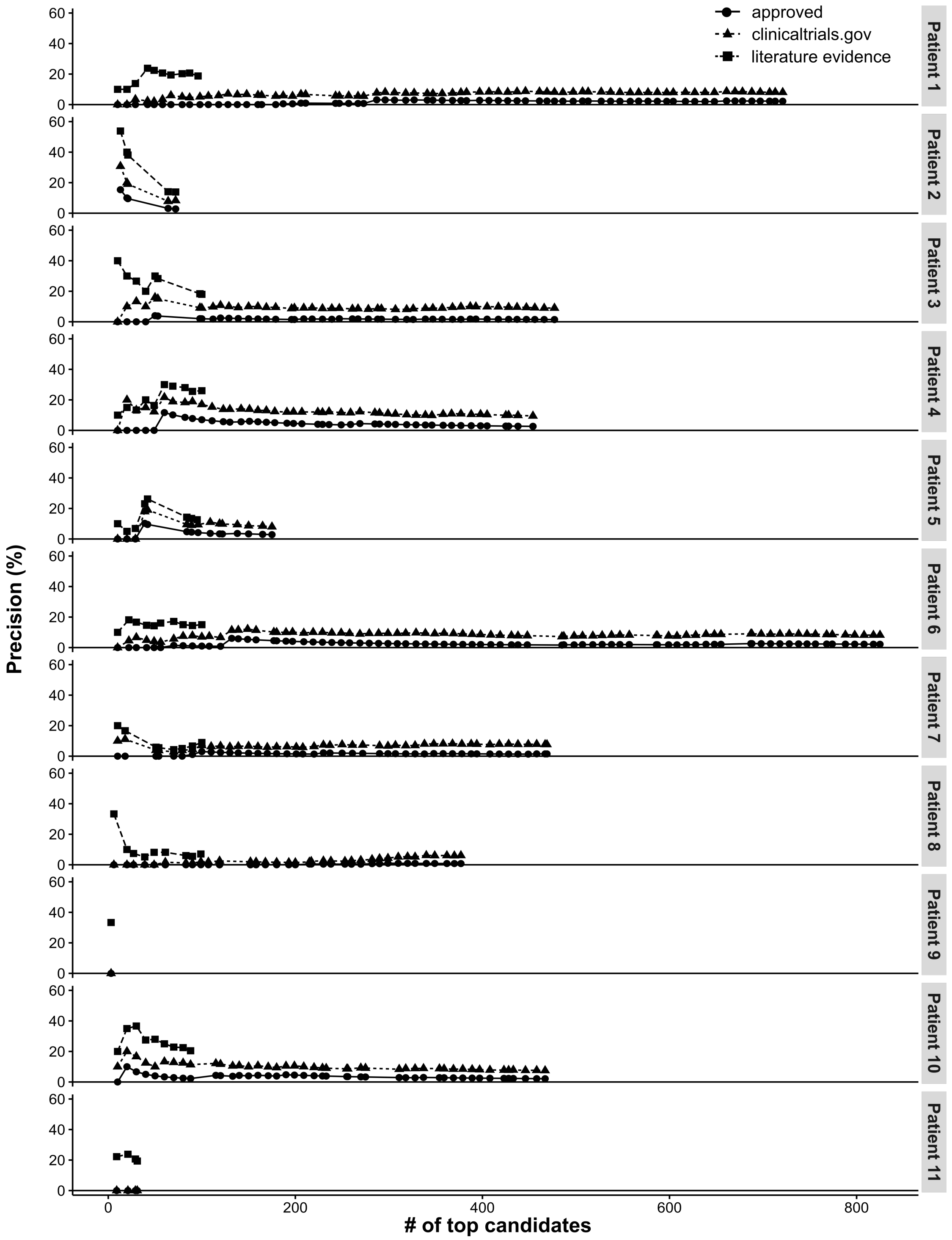

Fig. S13. Precision among ranked candidates of all individual CD patients. Drug rank on the x-axis and precision on the y-axis. Showing precision for approved CD drugs, drugs that were registered for clinical trials in CD as well as for drugs with literature evidence as indicated by shape and line type. Literature evidence was only collected for candidates with a rank up to 100.

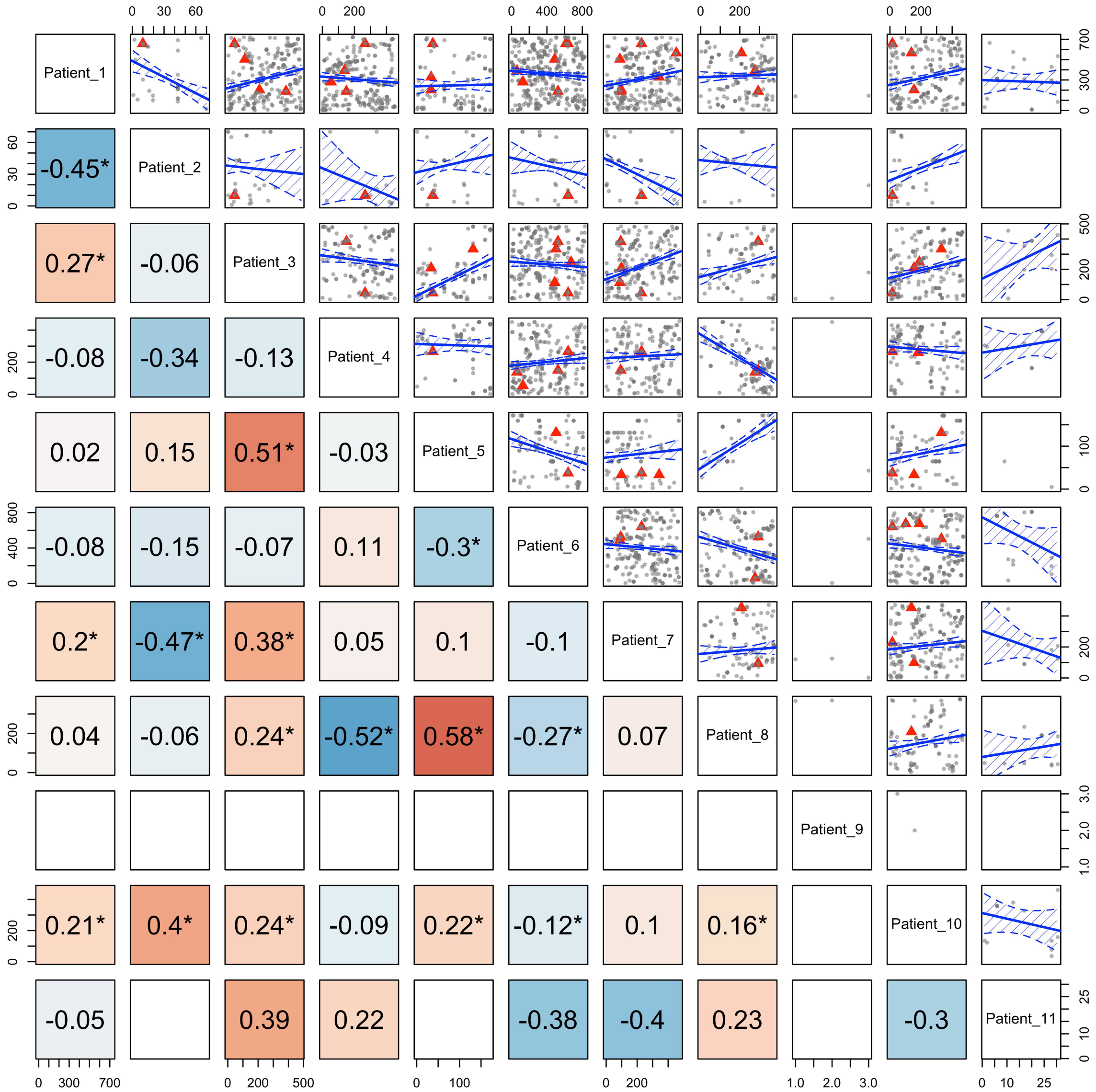

Fig. S14. Correlation between individual Crohn's disease patient drug ranks. Patients are indicated on the diagonal axis. The upper right portion of this panel presents scatter plots depicting the correlation of drug ranks in drug predictions from two individual patients. Known CD drugs are depicted by red triangles, and all other drugs are presented as gray dots. In cases where more than 5 drugs overlapped between patients, a Pearson correlation was calcuated. The correlation coefficients are presented in the lower left panel and are coloured by correlation coefficient (0 > blue and red > 0), and significant P values are indicated by asterisks. Blue lines in the upper right panel show the Pearson correlation coefficient and 95% confidence intervals.

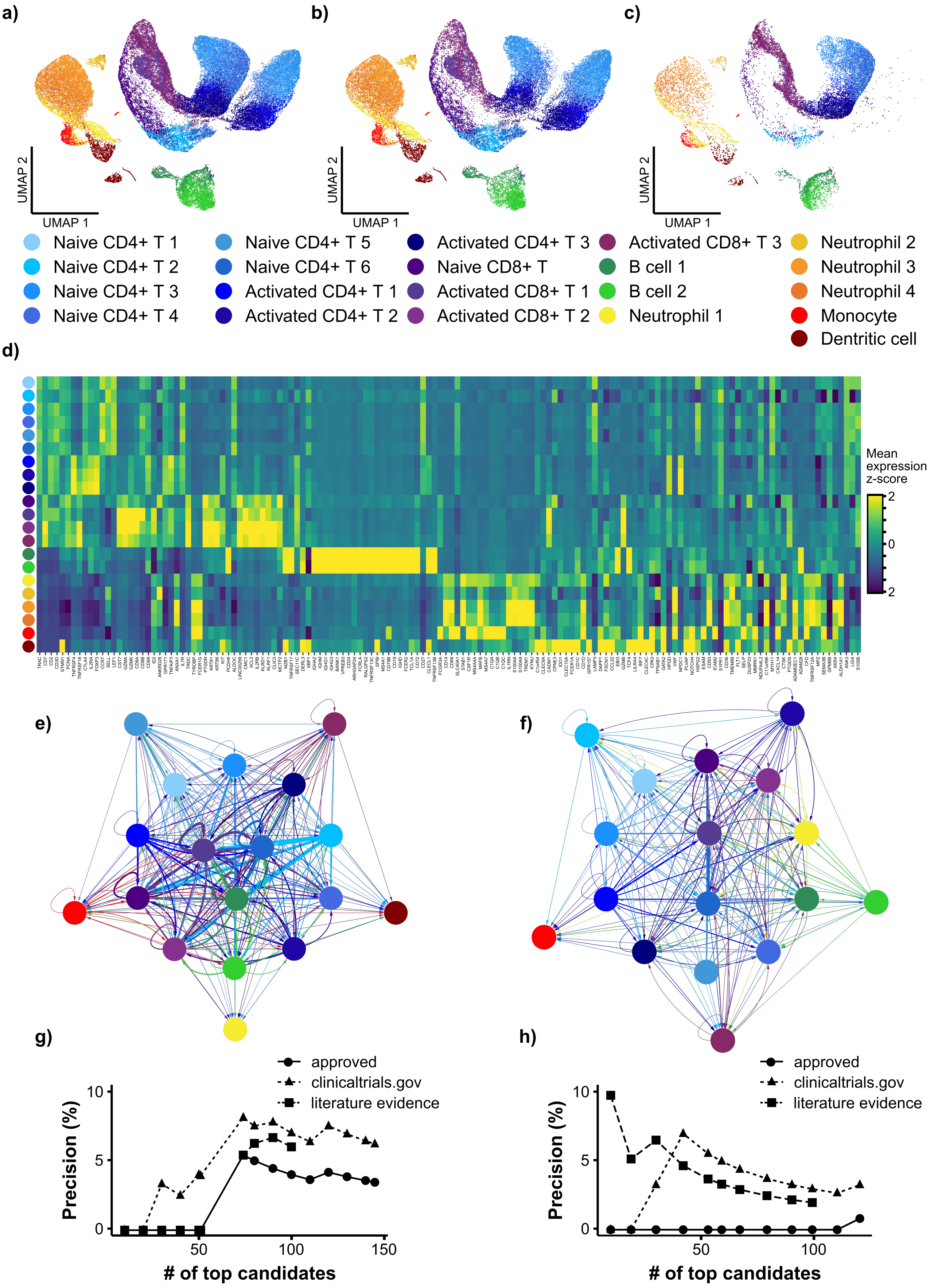
a)
b)
c)
UMAP 2
UMAP 1
Naive CD4+ T 1
Naive CD4+ T 2
Naive CD4+ T 3
Naive CD4+ T 4
Naive CD4+ T 5
Naive CD4+ T 6
Activated CD4+ T 1
Activated CD4+ T 2
Activated CD4+ T 3
Naive CD8+ T
Activated CD8+ T 1
Activated CD8+ T 2
Activated CD8+ T 3
B cell 1
B cell 2
Neutrophil 1
Neutrophil 2
Neutrophil 3
Neutrophil 4
Monocyte
Dentritic cell
d)
Mean expression z-score
2
0
-2
e)
f)
g)
h)
approved
clinicaltrials.gov
literature evidence
Precision (%)
# of top candidates
0
5
10
50
100
150

Fig. S15. scDrugPrio applied to psoriatic arthritis patients who were or were not anti-IL17 responders. UMAP visualisation of cells from **a)** controls and PsA patients (both responders and nonresponders to anti-IL17), **b)** only PsA patients, and **c)** only controls. **d)** Cell typing performed on marker genes; cell type is indicated by cluster colours to the left. MCDMs were created for **e)** anti-IL17 responders and **f)** nonresponders. Precision for approved PsA drugs, drugs for which clincial trials had been registered in PsA and drugs with literature evidence (only collected for top 100 ranking drugs) for **g)** responders and **h)** nonresponders were derived separately. Responders achieve a precision of 4% for approved PsA drugs and 6% for drugs with literature evidence among the top 100 ranking drugs. The approved PsA drugs are cortisone derivates. Nonresponders have a precision of 0% for approved PsA drugs and 2% for drugs with literature evidence among the top 100 ranking drugs.

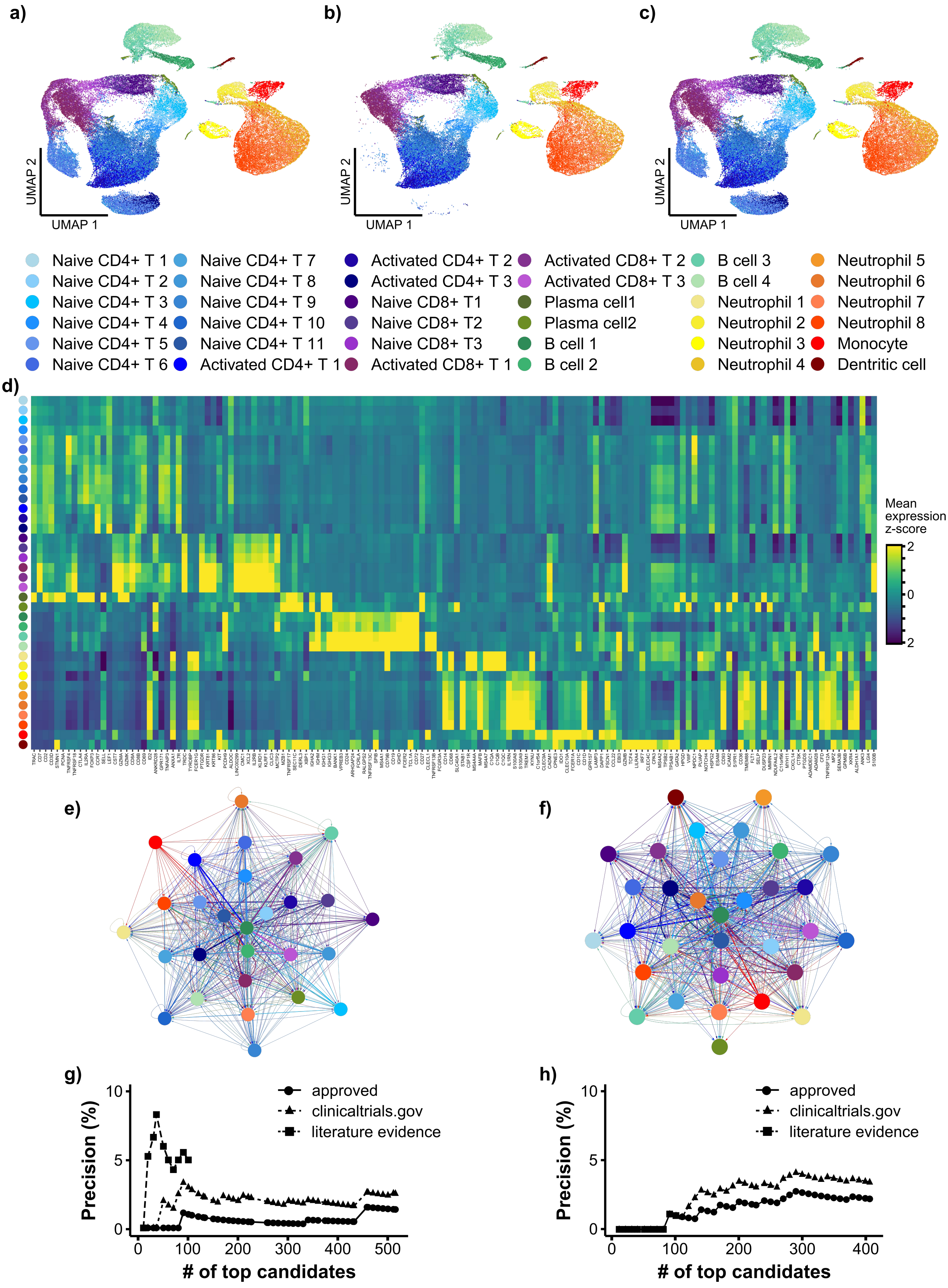
a)
b)
c)
UMAP 2
UMAP 1
Naive CD4+ T 1
Naive CD4+ T 2
Naive CD4+ T 3
Naive CD4+ T 4
Naive CD4+ T 5
Naive CD4+ T 6
Naive CD4+ T 7
Naive CD4+ T 8
Naive CD4+ T 9
Naive CD4+ T 10
Naive CD4+ T 11
Activated CD4+ T 1
Activated CD4+ T 2
Activated CD4+ T 3
Naive CD8+ T1
Naive CD8+ T2
Naive CD8+ T3
Activated CD8+ T 1
Activated CD8+ T 2
Activated CD8+ T 3
Plasma cell1
Plasma cell2
B cell 1
B cell 2
B cell 3
B cell 4
Neutrophil 1
Neutrophil 2
Neutrophil 3
Neutrophil 4
Neutrophil 5
Neutrophil 6
Neutrophil 7
Neutrophil 8
Monocyte
Dentritic cell
d)
Mean expression z-score
2
0
-2
e)
f)
g)
h)
approved
clinicaltrials.gov
literature evidence
Precision (%)
# of top candidates

Fig. S16. scDrugPrio applied to psoriatic arthritis patients who were or were not anti-TNF responders. UMAP visualisation of cells from **a)** controls and PsA patients (both responders and nonresponders to anti-TNF), **b)** only PsA patients, and **c)** only controls. **d)** Cell typing performed on marker genes; cell type is indicated by cluster colours to the left. MCDMs were created for **e)** anti-TNF responders and **f)** nonresponders. Precision for approved PsA drugs, drugs for which clincial trials had been registered in PsA and drugs with literature evidence (only collected for top 100 ranking drugs) for **g)** responders and **h)** nonresponders were derived separately. Responders achieve a precision of 1% for approved PsA drugs and 4% for drugs with literature evidence among the top 100 ranking drugs. The approved PsA drugs are cortisone derivates. Nonresponders have a precision of 1% for approved PsA drugs and 1% for drugs with literature evidence among the top 100 ranking drugs.

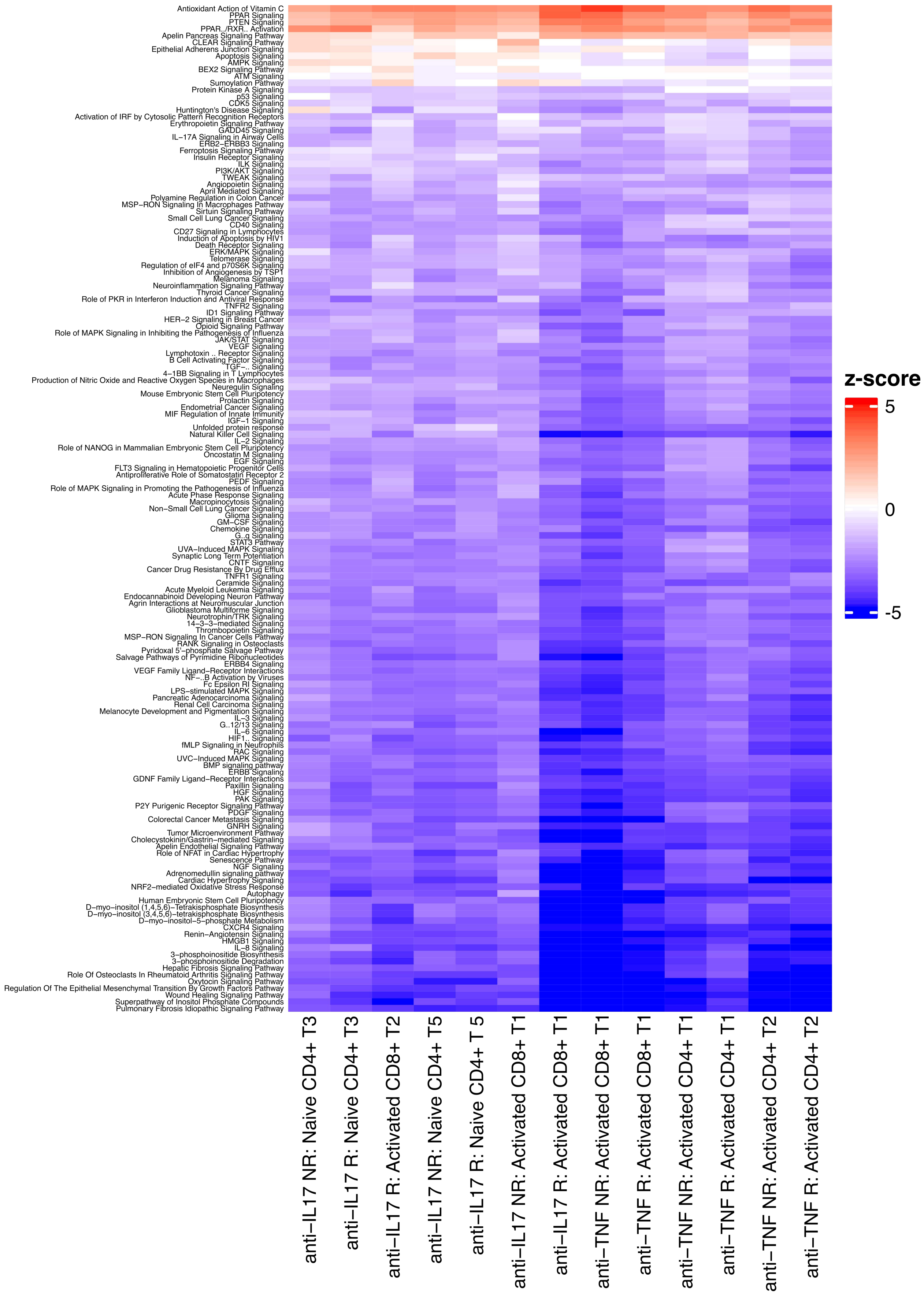

Antioxidant Action of Vitamin C
PPAR Signaling
PTEN Signaling
PPAR../RXR.. Activation
Apelin Pancreas Signaling Pathway
CLEAR Signaling Pathway
Epithelial Adherens Junction Signaling
Apoptosis Signaling
AMPK Signaling
BEX2 Signaling Pathway
ATM Signaling
Sumoylation Pathway
Protein Kinase A Signaling
p53 Signaling
CDK5 Signaling
Huntington's Disease Signaling
Activation of IRF by Cytosolic Pattern Recognition Receptors
Erythropoietin Signaling Pathway
GADD45 Signaling
IL−17A Signaling in Airway Cells
ERB2−ERBB3 Signaling
Ferroptosis Signaling Pathway
Insulin Receptor Signaling
ILK Signaling
PI3K/AKT Signaling
TWEAK Signaling
Angiopoietin Signaling
April Mediated Signaling
Polyamine Regulation in Colon Cancer
MSP−RON Signaling In Macrophages Pathway
Sirtuin Signaling Pathway
Small Cell Lung Cancer Signaling
CD40 Signaling
CD27 Signaling in Lymphocytes
Induction of Apoptosis by HIV1
Death Receptor Signaling
ERK/MAPK Signaling
Telomerase Signaling
Regulation of eIF4 and p70S6K Signaling
Inhibition of Angiogenesis by TSP1
Melanoma Signaling
Neuroinflammation Signaling Pathway
Thyroid Cancer Signaling
Role of PKR in Interferon Induction and Antiviral Response
TNFR2 Signaling
ID1 Signaling Pathway
HER−2 Signaling in Breast Cancer
Opioid Signaling Pathway
Role of MAPK Signaling in Inhibiting the Pathogenesis of Influenza
JAK/STAT Signaling
VEGF Signaling
Lymphotoxin .. Receptor Signaling
B Cell Activating Factor Signaling
TGF−.. Signaling
4−1BB Signaling in T Lymphocytes
Production of Nitric Oxide and Reactive Oxygen Species in Macrophages
Neuregulin Signaling
Mouse Embryonic Stem Cell Pluripotency
Prolactin Signaling
Endometrial Cancer Signaling
MIF Regulation of Innate Immunity
IGF−1 Signaling
Unfolded protein response
Natural Killer Cell Signaling
IL−2 Signaling
Role of NANOG in Mammalian Embryonic Stem Cell Pluripotency
Oncostatin M Signaling
EGF Signaling
FLT3 Signaling in Hematopoietic Progenitor Cells
Antiproliferative Role of Somatostatin Receptor 2
PEDF Signaling
Role of MAPK Signaling in Promoting the Pathogenesis of Influenza
Acute Phase Response Signaling
Macropinocytosis Signaling
Non−Small Cell Lung Cancer Signaling
Glioma Signaling
GM−CSF Signaling
Chemokine Signaling
G..q Signaling
STAT3 Pathway
UVA−Induced MAPK Signaling
Synaptic Long Term Potentiation
CNTF Signaling
Cancer Drug Resistance By Drug Efflux
TNFR1 Signaling
Ceramide Signaling
Acute Myeloid Leukemia Signaling
Endocannabinoid Developing Neuron Pathway
Agrin Interactions at Neuromuscular Junction
Glioblastoma Multiforme Signaling
Neurotrophin/TRK Signaling
14−3−3−mediated Signaling
Thrombopoietin Signaling
MSP−RON Signaling In Cancer Cells Pathway
RANK Signaling in Osteoclasts
Pyridoxal 5'−phosphate Salvage Pathway
Salvage Pathways of Pyrimidine Ribonucleotides
ERBB4 Signaling
VEGF Family Ligand−Receptor Interactions
NF−..B Activation by Viruses
Fc Epsilon RI Signaling
LPS−stimulated MAPK Signaling
Pancreatic Adenocarcinoma Signaling
Renal Cell Carcinoma Signaling
Melanocyte Development and Pigmentation Signaling
IL−3 Signaling
G..12/13 Signaling
IL−6 Signaling
HIF1.. Signaling
fMLP Signaling in Neutrophils
RAC Signaling
UVC−Induced MAPK Signaling
BMP signaling pathway
ERBB Signaling
GDNF Family Ligand−Receptor Interactions
Paxillin Signaling
HGF Signaling
PAK Signaling
P2Y Purigenic Receptor Signaling Pathway
PDGF Signaling
Colorectal Cancer Metastasis Signaling
GNRH Signaling
Tumor Microenvironment Pathway
Cholecystokinin/Gastrin−mediated Signaling
Apelin Endothelial Signaling Pathway
Role of NFAT in Cardiac Hypertrophy
Senescence Pathway
NGF Signaling
Adrenomedullin signaling pathway
Cardiac Hypertrophy Signaling
NRF2−mediated Oxidative Stress Response
Autophagy
Human Embryonic Stem Cell Pluripotency
D−myo−inositol (1,4,5,6)−Tetrakisphosphate Biosynthesis
D−myo−inositol (3,4,5,6)−tetrakisphosphate Biosynthesis
D−myo−inositol−5−phosphate Metabolism
CXCR4 Signaling
Renin−Angiotensin Signaling
HMGB1 Signaling
IL−8 Signaling
3−phosphoinositide Biosynthesis
3−phosphoinositide Degradation
Hepatic Fibrosis Signaling Pathway
Role Of Osteoclasts In Rheumatoid Arthritis Signaling Pathway
Oxytocin Signaling Pathway
Regulation Of The Epithelial Mesenchymal Transition By Growth Factors Pathway
Wound Healing Signaling Pathway
Superpathway of Inositol Phosphate Compounds
Pulmonary Fibrosis Idiopathic Signaling Pathway
anti−IL17 NR: Naive CD4+ T3
anti−IL17 R: Naive CD4+ T3
anti−IL17 R: Activated CD8+ T2
anti−IL17 NR: Naive CD4+ T5
anti−IL17 R: Naive CD4+ T 5
anti−IL17 NR: Activated CD8+ T1
anti−IL17 R: Activated CD8+ T1
anti−TNF NR: Activated CD8+ T1
anti−TNF R: Activated CD8+ T1
anti−TNF NR: Activated CD4+ T1
anti−TNF R: Activated CD4+ T1
anti−TNF NR: Activated CD4+ T2
anti−TNF R: Activated CD4+ T2
z-score
5
0
-5

Fig. S17. Ingenuity pathway analysis of cell types enriching IL17 or TNF KEGG pathways. To investigate why scDrugPrio does not prioritize valid targets based on PBMC data from PsA patients, we checked whether treatment-relevant pathways were enriched among the DEGs of responders and nonresponders to the respective drug. We found that only a few cell types were enriched in the TNF-α and IL17 KEGG pathways. Since KEGG does not enable prediction of up- or downregulation, we redid pathway enrichment for the relevant cell types in ingenuity pathway analysis (IPA). In this heatmap, we present IPA-derived z-scores for up- (positive) or down (negative) pathway regulation. The IL17A signalling pathway and TNFR signalling pathway were either downregulated or insignificantly enriched, with a z-score trending towards downregulation.

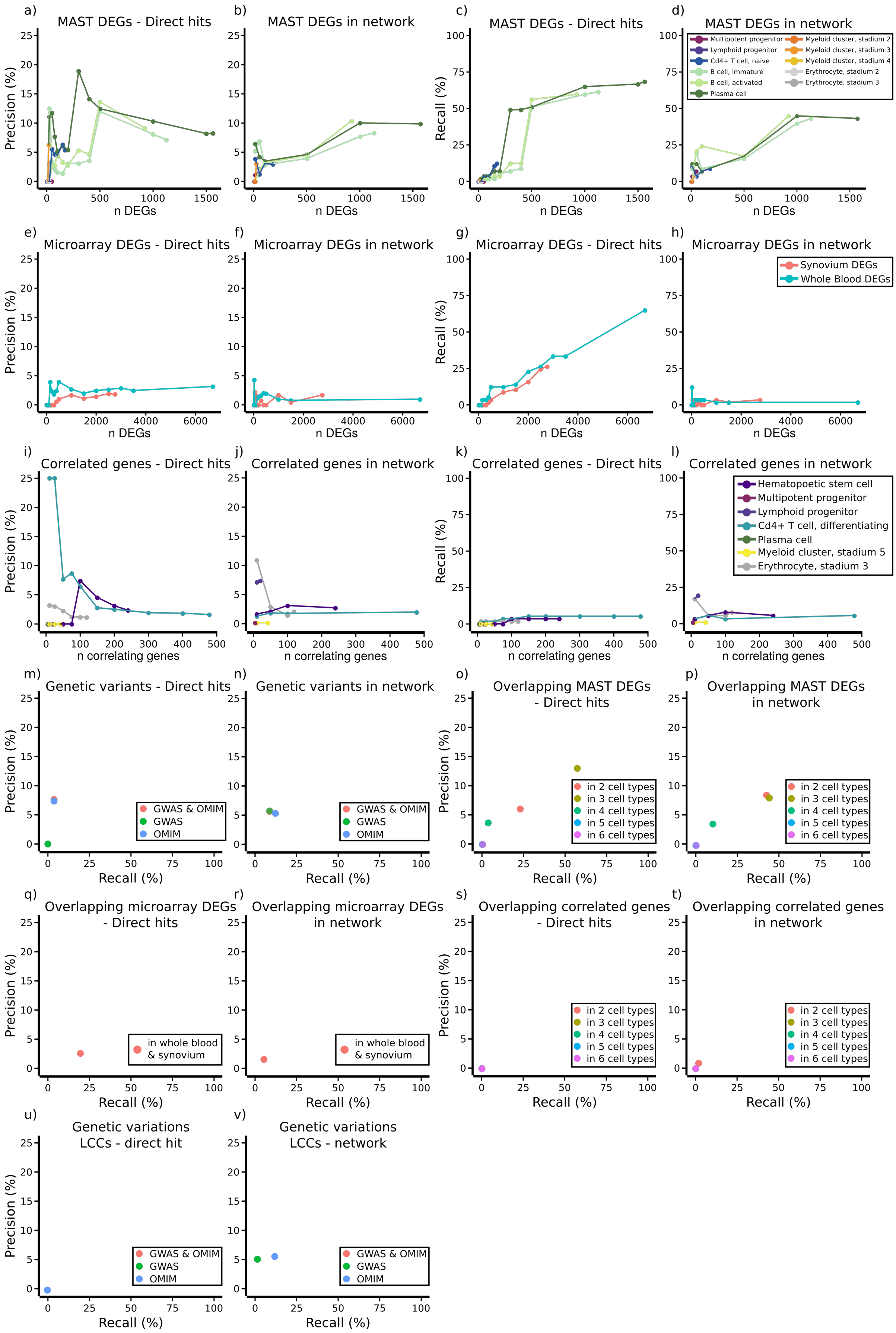

a)
MAST DEGs - Direct hits
Precision (%)
n DEGs
b)
MAST DEGs in network
n DEGs
c)
MAST DEGs - Direct hits
Recall (%)
n DEGs
d)
MAST DEGs in network
Multipotent progenitor
Lymphoid progenitor
Cd4+ T cell, naive
B cell, immature
B cell, activated
Plasma cell
Myeloid cluster, stadium 2
Myeloid cluster, stadium 3
Myeloid cluster, stadium 4
Erythrocyte, stadium 2
Erythrocyte, stadium 3
n DEGs
e)
Microarray DEGs - Direct hits
Precision (%)
n DEGs
f)
Microarray DEGs in network
n DEGs
g)
Microarray DEGs - Direct hits
Recall (%)
n DEGs
h)
Microarray DEGs in network
Synovium DEGs
Whole Blood DEGs
n DEGs
i)
Correlated genes - Direct hits
Precision (%)
n correlating genes
j)
Correlated genes in network
n correlating genes
k)
Correlated genes - Direct hits
Recall (%)
n correlating genes
l)
Correlated genes in network
Hematopoetic stem cell
Multipotent progenitor
Lymphoid progenitor
Cd4+ T cell, differentiating
Plasma cell
Myeloid cluster, stadium 5
Erythrocyte, stadium 3
n correlating genes
m)
Genetic variants - Direct hits
Precision (%)
GWAS & OMIM
GWAS
OMIM
Recall (%)
n)
Genetic variants in network
GWAS & OMIM
GWAS
OMIM
Recall (%)
o)
Overlapping MAST DEGs
- Direct hits
Precision (%)
in 2 cell types
in 3 cell types
in 4 cell types
in 5 cell types
in 6 cell types
Recall (%)
p)
Overlapping MAST DEGs
in network
in 2 cell types
in 3 cell types
in 4 cell types
in 5 cell types
in 6 cell types
Recall (%)
q)
Overlapping microarray DEGs
- Direct hits
Precision (%)
in whole blood
& synovium
Recall (%)
r)
Overlapping microarray DEGs
in network
in whole blood
& synovium
Recall (%)
s)
Overlapping correlated genes
- Direct hits
Precision (%)
in 2 cell types
in 3 cell types
in 4 cell types
in 5 cell types
in 6 cell types
Recall (%)
t)
Overlapping correlated genes
in network
in 2 cell types
in 3 cell types
in 4 cell types
in 5 cell types
in 6 cell types
Recall (%)
u)
Genetic variations
LCCs - direct hit
Precision (%)
GWAS & OMIM
GWAS
OMIM
Recall (%)
v)
Genetic variations
LCCs - network
GWAS & OMIM
GWAS
OMIM
Recall (%)

Fig. S18 Precision and recall as a function of different feature selection methods and different data sets. a-d) Precision (a, b) and recall (c, d) derived based on the top x most significant MAST DEGs. Selected genes were checked for known drug targets, and drugs that targeted at least one DEG ("Direct hits") were selected as candidates for calculation of precision (a) and recall (c). Selected genes were also used as input to the previously described network-proximity screening, and drugs that showed $z_c < -1.64$ in the literature curated PPIN were selected as candidates for calculation of precision (b) and recall (d). **e-h)** correspond to a-b) for microarray-derived DEGs. **i-l)** corresponds to a-b) for genes derived by correlation of each cell type's gene expression values with the arthritis score of the AIA mice. **m&n)** shows precision/recall plots for GWAS genes and OMIM genes (as previously defined) as well as a combined gene list. **o&p)** Precision/recall plot investigating the predictive capability of MAST DEGs that overlapped between x cell types. **q&r)** corresponding to o&p) for overlapping microarray-derived DEGs. **s&t)** corresponding to o&p) for cell type-specific genes correlated with arthritis score. **u&v)** Precision/recall plot for the LCCs formed by genetic variations.

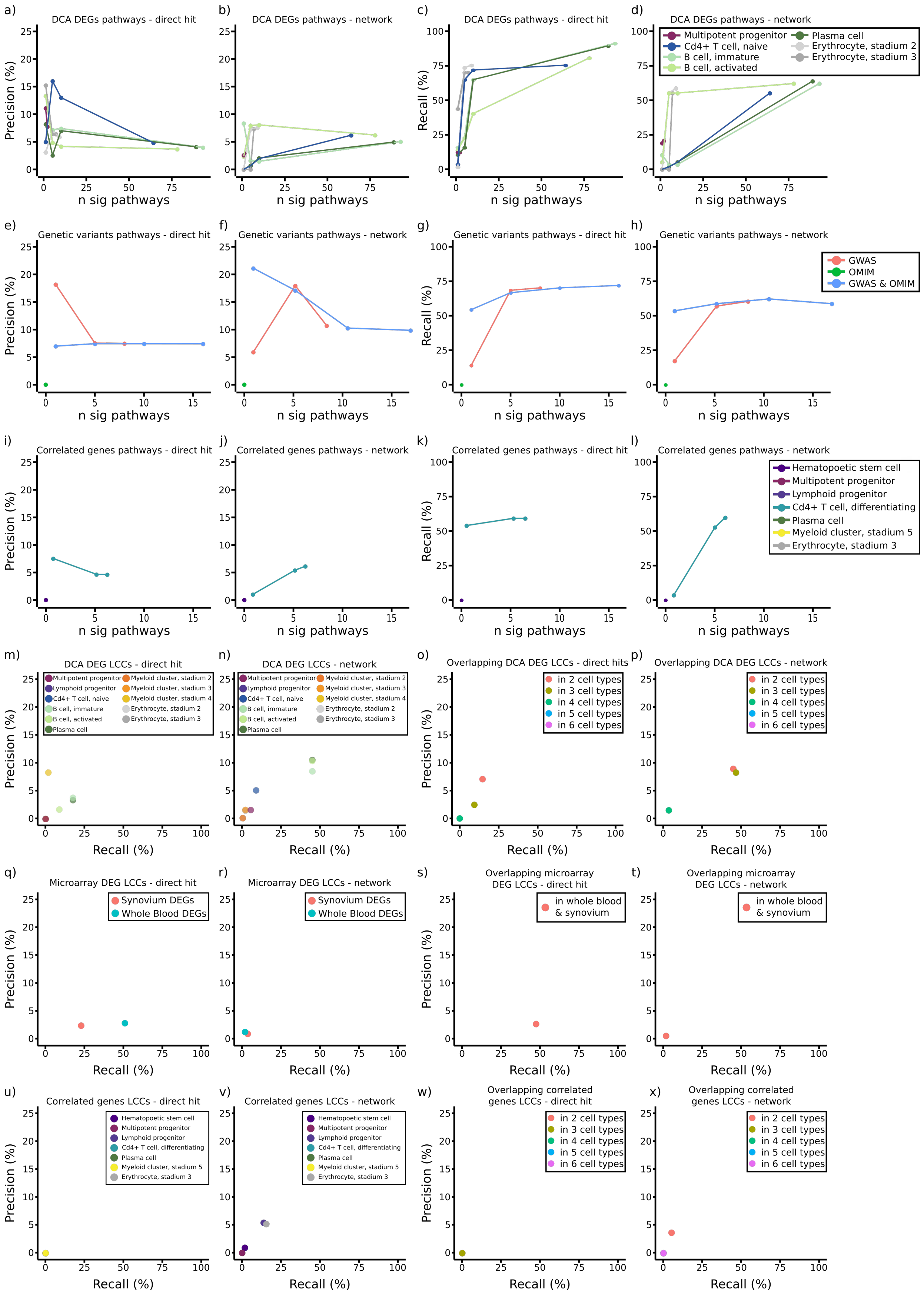
a)
DCA DEGs pathways - direct hit
b)
DCA DEGs pathways - network
c)
DCA DEGs pathways - direct hit
d)
DCA DEGs pathways - network
Multipotent progenitor
Cd4+ T cell, naive
B cell, immature
B cell, activated
Plasma cell
Erythrocyte, stadium 2
Erythrocyte, stadium 3
e)
Genetic variants pathways - direct hit
f)
Genetic variants pathways - network
g)
Genetic variants pathways - direct hit
h)
Genetic variants pathways - network
GWAS
OMIM
GWAS & OMIM
i)
Correlated genes pathways - direct hit
j)
Correlated genes pathways - network
k)
Correlated genes pathways - direct hit
l)
Correlated genes pathways - network
Hematopoetic stem cell
Multipotent progenitor
Lymphoid progenitor
Cd4+ T cell, differentiating
Plasma cell
Myeloid cluster, stadium 5
Erythrocyte, stadium 3
Precision (%)
Recall (%)
n sig pathways
m)
DCA DEG LCCs - direct hit
n)
DCA DEG LCCs - network
Multipotent progenitor
Lymphoid progenitor
Cd4+ T cell, naive
B cell, immature
B cell, activated
Plasma cell
Myeloid cluster, stadium 2
Myeloid cluster, stadium 3
Myeloid cluster, stadium 4
Erythrocyte, stadium 2
Erythrocyte, stadium 3
o)
Overlapping DCA DEG LCCs - direct hits
p)
Overlapping DCA DEG LCCs - network
in 2 cell types
in 3 cell types
in 4 cell types
in 5 cell types
in 6 cell types
q)
Microarray DEG LCCs - direct hit
r)
Microarray DEG LCCs - network
Synovium DEGs
Whole Blood DEGs
s)
Overlapping microarray DEG LCCs - direct hit
t)
Overlapping microarray DEG LCCs - network
in whole blood & synovium
u)
Correlated genes LCCs - direct hit
v)
Correlated genes LCCs - network
Hematopoetic stem cell
Multipotent progenitor
Lymphoid progenitor
Cd4+ T cell, differentiating
Plasma cell
Myeloid cluster, stadium 5
Erythrocyte, stadium 3
w)
Overlapping correlated genes LCCs - direct hit
x)
Overlapping correlated genes LCCs - network
in 2 cell types
in 3 cell types
in 4 cell types
in 5 cell types
in 6 cell types
Precision (%)
Recall (%)

Fig S19. Precision and recall as a function of different feature selection methods and different data sets. Continuation of **Fig. S8**. **a-d)** Precision (a, b) and recall (c, d) derived based on the top significantly single-cell derived DEG-enriched KEGG pathways. Selected genes were checked for known drug targets, and drugs that targeted at least one DEG ("Direct hits") were selected as candidates for calculation of precision (a) and recall (c). Selected genes were also used as input to the previously described network-proximity screening, and drugs that showed $z_c < -1.64$ in the literature curated PPIN were selected as candidates for calculation of precision (b) and recall (d). **e-h)** corresponds to a-b) for genetic variations. **i-l)** corresponds to a-b) for genes derived by correlation of each cell type's gene expression values with the arthritis score of the AIA mice. **m&n)** Precision/recall plots for LCCs formed by single-cell derived DEGs. **o&p)** Precision/recall plot for the overlap between LCCs formed by single-cell derived cell type-specific DEGs. **q&r)** corresponding to m&n) for microarray-derived DEGs. **s&t)** corresponding to o&p) for LCCs formed by microarray-derived DEGs. **u&v)** corresponding to m&n) for LCCs formed by cell type-specific genes that correlated with arthritis severity score. **w&x)** corresponding to o&p) for genes overlapping between LCCs formed by correlated genes.

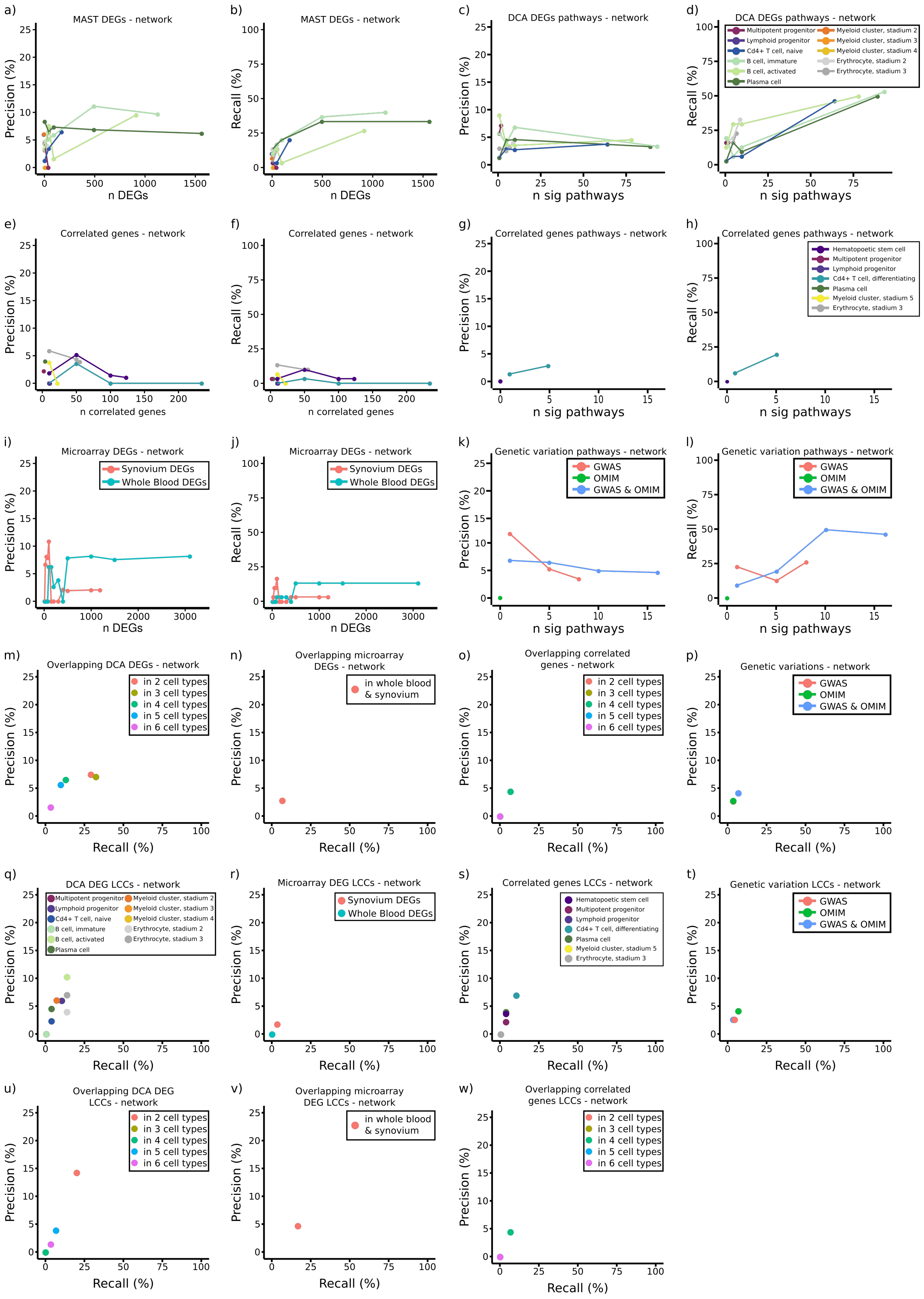
a)
MAST DEGs - network
Precision (%)
n DEGs
b)
MAST DEGs - network
Recall (%)
n DEGs
c)
DCA DEGs pathways - network
Precision (%)
n sig pathways
d)
DCA DEGs pathways - network
Multipotent progenitor
Lymphoid progenitor
Cd4+ T cell, naive
B cell, immature
B cell, activated
Plasma cell
Myeloid cluster, stadium 2
Myeloid cluster, stadium 3
Myeloid cluster, stadium 4
Erythrocyte, stadium 2
Erythrocyte, stadium 3
Recall (%)
n sig pathways
e)
Correlated genes - network
Precision (%)
n correlated genes
f)
Correlated genes - network
Recall (%)
n correlated genes
g)
Correlated genes pathways - network
Precision (%)
n sig pathways
h)
Correlated genes pathways - network
Hematopoetic stem cell
Multipotent progenitor
Lymphoid progenitor
Cd4+ T cell, differentiating
Plasma cell
Myeloid cluster, stadium 5
Erythrocyte, stadium 3
Recall (%)
n sig pathways
i)
Microarray DEGs - network
Synovium DEGs
Whole Blood DEGs
Precision (%)
n DEGs
j)
Microarray DEGs - network
Synovium DEGs
Whole Blood DEGs
Recall (%)
n DEGs
k)
Genetic variation pathways - network
GWAS
OMIM
GWAS & OMIM
Precision (%)
n sig pathways
l)
Genetic variation pathways - network
GWAS
OMIM
GWAS & OMIM
Recall (%)
n sig pathways
m)
Overlapping DCA DEGs - network
in 2 cell types
in 3 cell types
in 4 cell types
in 5 cell types
in 6 cell types
Precision (%)
Recall (%)
n)
Overlapping microarray DEGs - network
in whole blood & synovium
Precision (%)
Recall (%)
o)
Overlapping correlated genes - network
in 2 cell types
in 3 cell types
in 4 cell types
in 5 cell types
in 6 cell types
Precision (%)
Recall (%)
p)
Genetic variations - network
GWAS
OMIM
GWAS & OMIM
Precision (%)
Recall (%)
q)
DCA DEG LCCs - network
Multipotent progenitor
Lymphoid progenitor
Cd4+ T cell, naive
B cell, immature
B cell, activated
Plasma cell
Myeloid cluster, stadium 2
Myeloid cluster, stadium 3
Myeloid cluster, stadium 4
Erythrocyte, stadium 2
Erythrocyte, stadium 3
Precision (%)
Recall (%)
r)
Microarray DEG LCCs - network
Synovium DEGs
Whole Blood DEGs
Precision (%)
Recall (%)
s)
Correlated genes LCCs - network
Hematopoetic stem cell
Multipotent progenitor
Lymphoid progenitor
Cd4+ T cell, differentiating
Plasma cell
Myeloid cluster, stadium 5
Erythrocyte, stadium 3
Precision (%)
Recall (%)
t)
Genetic variation LCCs - network
GWAS
OMIM
GWAS & OMIM
Precision (%)
Recall (%)
u)
Overlapping DCA DEG LCCs - network
in 2 cell types
in 3 cell types
in 4 cell types
in 5 cell types
in 6 cell types
Precision (%)
Recall (%)
v)
Overlapping microarray DEG LCCs - network
in whole blood & synovium
Precision (%)
Recall (%)
w)
Overlapping correlated genes LCCs - network
in 2 cell types
in 3 cell types
in 4 cell types
in 5 cell types
in 6 cell types
Precision (%)
Recall (%)

Fig S20. Precision and recall in the HuRI protein–protein interaction network (PPIN) as a function of different feature selection methods and different data sets. For calculation of the above plots, genes that were not found in the HuRI PPIN were removed prior to calculation. This included even the removal of drugs from the calculation if they did not target any gene included in HuRI. This panel exclusively shows the network-proximity screening outcomes in the HuRI PPIN, and drugs that showed $z_c < -1.64$ were selected as candidates for calculation of precision (b) and recall (d). **a&b)** Precision and recall for the top x most significantly single-cell derived DEGs. **c&d)** Precision and recall for DCA DEG-enriched KEGG pathways. **e&f)** corresponding to a&b) for correlated genes. **g&h)** corresponding to c&d) with correlated gene enriched KEGG pathways. **i&j)** corresponding to **a&b)** for microarray-derived DEGs. **k&l)** corresponding to c&d) for genetic variation-enriched KEGG pathways. **m-o)** Precision/recall plot for overlapping single-cell derived DEGs, microarray DEGs and correlated genes, respectively. **p)** Precision/recall plot for genetic variations. **q-t)** Precision/recall plots for LCCs formed by single-cell derived DEGs, microarray DEGs, genes correlated with arthritis score, and genetic variations. **u-w)** Precision/recall plots for LCCs overlapping between cell type-specific single-cell derived DEGs, microarray data sets, and cell type-specific genes correlated with arthritis severity score.

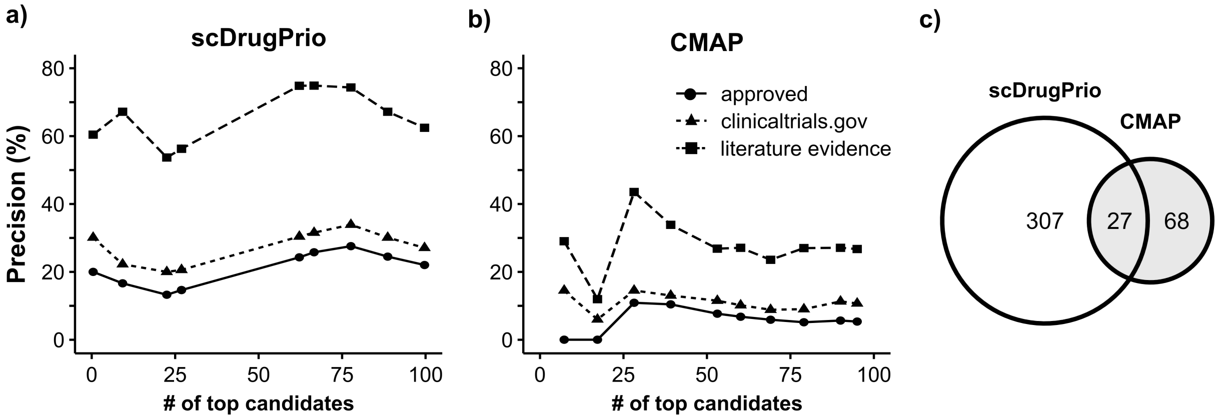

Fig. S21. Comparison of drug prediction using scDrugPrio and CMAP. Precision for **a)** the top 100 ranked candidates using scDrugPrio on DEGs derived from scRNA-seq AIA data. In a similar manner, **b)** CMAP prediction was conducted based on DEGs derived from pseudobulk RNA-seq of the AIA data. CMAP prediction was ranked by the CMAP effect measure. Precision is calculated for approved RA drugs, drugs for which clinical trials had been registered for RA and drugs with literature evidence among ranked drug candidates. Both drug predictions used the same DrugBank-derived drug target data for n = 1,840 drugs. **c)** shows the overlap between drug candidates.

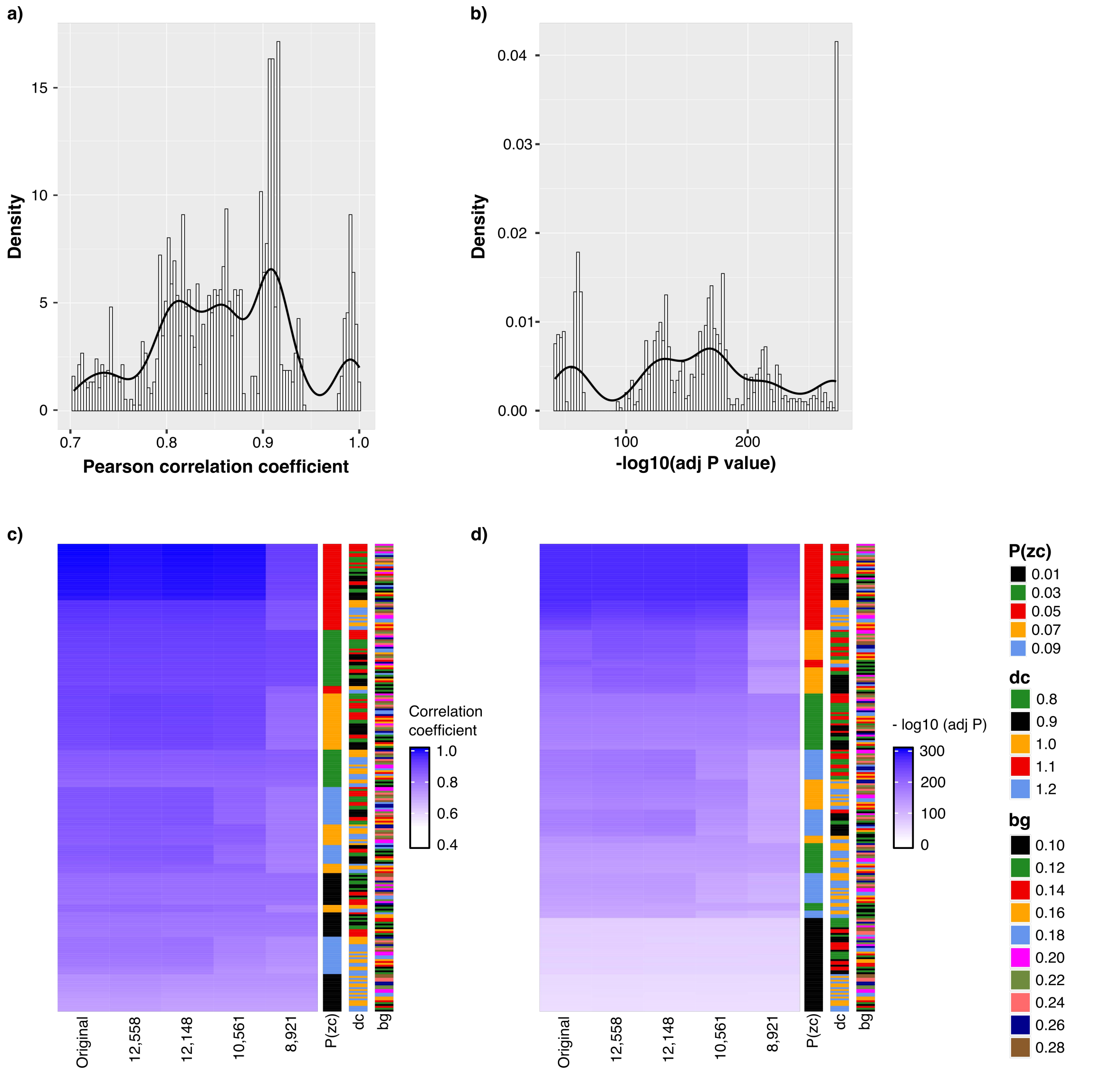

Fig. S22. Robustness analysis of scDrugPrio. Robustness was validated by variation of thresholds during recomputation of drug selection and drug ranking for the AIA data set. In this analysis, we varied 1) the number of DEGs that entered network calculations, 2) the P value cut-off for network proximity, derived from z_c, 3) network distance cut-off d_c, and 4) the cut-off for background gene calculation during NicheNet ligand activity analysis. Standard cut-offs for these included 1) all DEGs (n = 12,769), 2) P < 0.05, 3) dc < 1, and 4) Ea(i) ≥ 0.2. Collectively, 1250 drug rankings with varying thresholds were calculated, and the results were compared to the original drug ranking by Pearson correlation of drug ranks between computations. **a)** shows the distribution of Pearson correlation coefficients, **b)** shows the distribution of Bonferroni adjusted P values. Similarly, **c & d)** visualize the Pearson correlation coefficient and adjusted P value, respectively. Side bars indicate the combination of cut-offs used for results in each row. scDrugPrio returns overall returns stable and comparable results over a variety of thresholds.

References

1. Guney E, Menche J, Vidal M, Barabasi AL. Network-based in silico drug efficacy screening. Nat Commun. 2016;7:10331.
2. Eraslan G, Simon LM, Mircea M, Mueller NS, Theis FJ. Single-cell RNA-seq denoising using a deep count autoencoder. Nature communications. 2019;10(1):1-14.
3. Browaeys R, Saelens W, Saeys Y. NicheNet: modeling intercellular communication by linking ligands to target genes. Nature Methods. 2020;17(2):159-62.
