# Supplementary material for: scDrugPrio: a framework for the analysis of single-cell transcriptomics to address multiple problems in precision medicine in immune-mediated inflammatory diseases
Source: Genome Med. 2024 Mar 20;16:42. doi: 10.1186/s13073-024-01314-7 (PMC10956347; doi:10.1186/s13073-024-01314-7)
Supplement: Supplementary file 2 — Additional file 2: Supplement. Including supplementary methods and results as well as supplementary tables (Tables S1& S2). [file 13073_2024_1314_MOESM2_ESM.zip › Additional file 2 - Supplement.pdf]

scDrugPrio: A framework for the analysis of single-cell transcriptomics to address multiple problems in precision medicine in immune-mediated inflammatory diseases

Samuel Schäfer[1,2], Martin Smelik[3], Oleg Sysoev[4], Yelin Zhao[3], Desiré Eklund[1], Sandra Lilja[1,5], Mika Gustafsson[6], Holger Heyn[7,8], Antonio Julia[9], István A. Kovács[10,11], Joseph Loscalzo[12], Sara Marsal[9], Huan Zhang[1], Xinxiu Li[3], Danuta Gawel[5,¤], Hui Wang[3,13,¤], Mikael Benson[3,¤,*]

Affiliations

[1]Centre for Personalised Medicine, Linköping University; Linköping, Sweden.
[2]Department of Gastroenterology and Hepatology, University Hospital, Linköping, Sweden.
[3]Medical Digital Twin Research Group, Division of ENT, CLINTEC, Karolinska Institute, Stockholm, Sweden.
[4]Division of Statistics and Machine Learning, Department of Computer and Information Science, Linkoping University; Linköping, Sweden.
[5]Mavatar, Inc., Stockholm. Sweden
[6]Division for Bioinformatics, Department of Physics, Chemistry and Biology, Linköping University; Linköping, Sweden.
[7]CNAG-CRG, Centre for Genomic Regulation (CRG), Barcelona Institute of Science and Technology (BIST), 08028 Barcelona, Spain
[8]Universitat Pompeu Fabra (UPF), 08002 Barcelona, Spain
[9]Grup de Recerca de Reumatologia, Institut de Recerca Vall d'Hebron, Barcelona, España
[10]Department of Physics and Astronomy, Northwestern University, Evanston, IL 60208, USA.
[11]Northwestern Institute on Complex Systems, Northwestern University, Evanston, IL 60208, USA.
[12]Division of Cardiovascular Medicine, Channing Division of Network Medicine, Department of Medicine, Brigham and Women's Hospital, Harvard Medical School; Boston, MA, USA.
[13]Jiangsu Key Laboratory of Immunity and Metabolism, Department of Pathogenic Biology and Immunology, Xuzhou Medical University; Jiangsu, China.
[*]Corresponding author: mikael.benson@ki.se

Postal address: Mikael Benson, LIME/Medical Digital Twin Research Group, Karolinska Institute, Tomtebodavägen 18A. 171 65 Solna

[¤]Joint last authors.

Supplement

Identification of known disease-drug pairs based on DrugBank

Drug-disease associations were identified based on the "Indication" category in DrugBank. RA drugs were indicated for use in "Rheumatoid arthritis"/"RA". Drugs for Crohn's Disease were indicated for use in "Crohn's Disease", "IBD" and "Inflammatory Bowel Disease". Drugs for MS were selected based on the following DrugBank indications: "Active secondary progressive Multiple Sclerosis", "Progressive Multiple Sclerosis (PMS)", "Relapsing Remitting Multiple Sclerosis (RRMS)", "Progressive Relapsing Multiple Sclerosis", "Multiple sclerosis exacerbation", "Multiple Sclerosis, Primary Progressive", "Acute Multiple sclerosis", and "Relapsing Multiple Sclerosis (RMS)". PsA drugs were identified indicated for use on "Active Psoriatic arthritis", "Active Psoriatic arthritis", "Severe Psoriatic Arthritis", and "Psoriatic Arthritis".

The biological importance of the multicellular disease model of antigen-induced arthritis is supported by enrichment analyses

Since genetic variants have previously been associated with relevant disease mechanisms (1), gene set enrichment analysis was performed to evaluate the relevance of transcriptome-defined DEGs. Hence, for each cell type, a gene set enrichment was performed. To assess the relevance of the MCDM, we correlated eigenvector centralities of cell types in the MCDM and enrichment significance of GWAS gene enrichment among cell type-specific DEGs.

For this, the GWAS Catalog (2) version 1.0.2 was downloaded (January 2019), and GWAS genes were extracted for the following traits: "Celiac disease or Rheumatoid arthritis", "Rheumatoid arthritis", "Rheumatoid arthritis (ACPA-negative)", "Rheumatoid arthritis (ACPA-positive)", and "Rheumatoid arthritis (rheumatoid factor and/or anti-cyclic citrullinated peptide seropositive)". Intergenic SNPs were mapped towards the closest gene. Only GWAS genes with a genome-wide significance of $P < 10^{-8}$ were considered. GWAS gene enrichment among DEGs was calculated for each cluster separately with Fisher's exact test using genes with $Ea \geq 0.2$ (Eq. 1) as background genes. We found that intercellular cell type centrality correlated with GWAS enrichment significance.

The relevance of the inferred interactions in the MCDM were further supported by gene set enrichment analysis, which showed that known drug targets for human RA were significantly ($P < 0.05$) enriched among the ligands with the collectively most impactful interactions (as measured by summed Pearson correlation). Furthermore, we found that targets for US Food and Drug Administration (FDA)-approved RA drugs were enriched among DEGs of central cell types, such as immature and activated B cells (FDR adjusted $P < 0.01$ respectively; **Additional file 1: Fig. S2d-g**). Additionally, we performed a preranked gene set enrichment analysis (51) with the targets of RA drugs and the list of ligands ranked by the summed Pearson coefficient of all cell type interactions they were involved in as the input. We used 100,000 permutations, seed 149 and otherwise default parameters and found that RA drug targets were significantly ($P < 0.05$) enriched among the most impactful ligands (as measured by summed Pearson correlation).

Drug network degree supports intracellular centrality as a ranking measure

To test whether network centrality could be a potential proxy for drug effects, we examined the network degrees of all drugs included in DrugBank, as well as the degree of drugs that were approved for inflammatory diseases. For this analysis, we calculated the network degree of all genes included in the LCC of the literature-derived PPIN using the *degree()* function included in the igraph package (3). Next, we summarized the network degrees of drug targets. As many drugs had several targets, which complicated comparison to the background PPIN, we calculated 1) the max network degree of all targeted proteins, 2) the mean network degree, and 3) the sum of all targeted proteins network degrees (**Additional file 1: Fig. S3**). Drugs generally tended to target higher network degree targets. This observation became especially apparent when examining the network degree of drugs approved for the inflammatory diseases included in this study. Although antibody drugs target only a few proteins, they still tend to target proteins with higher network degrees. This is an interesting finding, as it could guide new drug target discovery strategies as well as spark additional research efforts aiming to examine why low network degree targets are underrepresented.

Intercellular and intracellular centrality for drug ranking

To evaluate intercellular centrality as a ranking measure in AIA, we performed a permutation test (1,000 iterations) that showed that the mean rank of established RA drugs was significantly better than the random expectation obtained from random permutations of intercellular centrality mean ranks (88.03 vs 168.07 ± 16.44; one-sided $P < 10^{-6)}$.

When ranking drugs only by intracellular centrality, the mean rank of established RA drugs was significantly better than random expectation (110.55 vs 167.20 ± 16.22; one-sided $P < 10^{-3}$), similar to the analysis of intercellular centrality above. To validate whether ranking based on both inter- and intracellular centrality held additional value, drugs were ranked primarily by combined intercellular centrality and second by intracellular centrality. When comparing the mean rank of RA drugs in this ranking to the random expectation obtained from 1,000 iterations in which drugs were ranked by intercellular centrality scores and randomly permuted intracellular centrality scores, we found that additional ordering according to mean intracellular centrality did not result in significantly better or worse ranking; however, we found that it acted as a tiebreaker for drugs achieving the same intercellular centrality-derived rank and increased precision for literature drugs among the top ranking drugs. We hence use a composite score derived from inter- and intracellular centrality for ranking.

Comparison of eigenvector centrality to other centralities

In the context of intercellular centrality, we calculated different centrality algorithms. The results can be found in **Additional file 3-7**. Different centrality approaches (e.g., eigenvector centralities, K-core decomposition, Kleinberg's hub centrality scores, Laplacian centrality, leverage centrality, group centrality) all rank the centrality of cell types similarly. This is exemplified by a high correlation between cell type eigenvector centrality scores and K-core decomposition (Pearson r = 0.77; $P < 10^{-3}$), Kleinberg's hub centrality scores (r = 0.72; $P < 0.01$), Laplacian centrality (r = 0.72; $P < 0.01$), leverage centrality (r = 0.85; $P < 10^{-4}$), and group centrality (r = 0.77; $P < 10^{-3}$). This comparison was performed in the AIA data set.

To test whether drug ranking using other centrality algorithms yielded similar results, we tested the above centrality algorithms that had yielded similar intercellular centralities. Comparison of results was based on Pearson correlation between drug ranks of eigenvector centrality-based results to that of drug ranks derived by other algorithms. Out of the above, we found that K-core decomposition (r = 0.80; $P < 10^{-300}$), Kleinberg's hub centrality scores (r = 0.86; $P < 10^{-300}$), Laplacian centrality (r = 0.86; $P < 10^{-300}$), and group centrality (r = 0.89; $P < 10^{-300}$) produced similar drug rankings (**Additional file 3**). This comparison was performed in the AIA data set.

Selection of the number of topmost significant DEGs used for network proximity calculation

As drug prioritization was dependent on the significance of network proximity (derived by bootstrap), high numbers of DEGs relative to the size of the network (literature PPIN LCC includes 17,651 unique proteins) result in $P(z_c) > 0.05$ for most drugs. Hence, a significance threshold was introduced to each data set individually that specified the number of topmost significant DEGs used in proximity calculations. To investigate where this threshold should be set, proximity calculations were performed over a range of different cut-offs (from a minimum of 100 DEGs up to all possible DEGs). The chosen threshold aimed to maximize precision and recall while simultaneously being consistent within a range of tried cut-offs. The network proximity calculations for MS patients and pooled CD patients were limited to the top 1,800 significant DEGs of each cell type. As these calculations consume immense computational resources and had initially been performed on non-batch-corrected data, batch-corrected data inherited their thresholds. For individual CD patient network proximity calculations, the threshold was set to the top 3,500 significant DEGs. For the PBMC data sets of PsA anti-TNF R and NR as well as PsA anti-IL17 R and NR, cut-offs were calculated on the batch-corrected data before network predictions were limited to the 3,000 top significant DEGs.

Validation of scDrugPrio compared to direct drug target identification and network predictions based on other disease genes.

To investigate the feasibility of developing a drug prioritization framework based on single-cell derived DEGs, we first tested whether the identified DEGs carried similar amounts of information

as other available gene sets, such as genetic variations (from GWAS Catalog (4) and OMIM (5)), microarray-derived (GSE55235 & GSE93272) (4) DEGs for rheumatoid arthritis, and genes for which cell type single-cell expression values correlated with clinical disease severity scores of the mice (referred to as correlated genes).

Furthermore, we evaluated different denoising strategies on the gene sets of interest aiming to condense and filter the gene set to derive better precision and recall for known RA drugs among the predicted drug candidates. Such denoising strategies included the use of 1) overlapping genes between gene sets (e.g., DEGs found in more than one cell type might arguably carry a higher predictive value), 2) the largest connected component (LCC) of a gene set, 3) Fisher pathway enrichment analysis using KEGG pathways (6), 4) the topmost significant genes in a gene set, and 5) combinations of these.

Drug candidate selection for the gene set of interest was attempted by 1) selecting all drugs that targeted at least one gene in the gene set of interest or by 2) applying the previously described network proximity-based approach (7) for which a cut-off of $z_c < -1.64$ was used (**Additional file 1: Fig. S18–S19**). All of the above predictions were repeated in the smaller, unbiased HuRI PPIN (8) (8236 proteins, 52150 interactions) to ensure the absence of knowledge bias (**Additional file 1: Fig. S20**).

Lastly, we benchmarked our approach to a preexisting approach (Connectivity-Map) developed for use on bulk RNA-seq data (**Additional file 1: Fig. S21**) and compared our results to results from a previous case report by Kim et al. in which scRNA-seq had been used to inform empirical drug choice for a patient who did not respond to standard treatment.

OMIM genes. We downloaded genes for “Rheumatoid arthritis” from the Online Mendelian Inheritance in Man (OMIM) database (5) (June 2019).

Microarray-derived DEGs. Microarray data for synovial tissue were downloaded from GEO (GSE55235) (9) and included data for healthy controls and RA patients. For further analysis, we

selected the synovial tissue samples of treated RA patients (n = 10) derived during joint replacement/synovectomy, as well as control samples that were obtained from postmortem joints (n = 10) of healthy individuals. Microarray data for whole blood samples of drug-naïve RA patients (n = 30) and healthy individuals (n = 30) were downloaded from GEO (GSE93272) (9, 10).

Data were downloaded utilizing the *getGEO()* function in the GEOquery R package (11). Microarray probes were translated to human Entrez Gene IDs utilizing the respective annotation files supplied by Affymetrix. If a probe ID corresponded to several Entrez Gene IDs, the probe was excluded from further analysis. If several probe IDs corresponded to the same Entrez Gene ID, only the probe ID with the highest fold change between sick and healthy samples was retained. DEG calculation was based on the expression values of the included samples and was conducted using the limma R package (12). First, a linear model for each gene's expression in all independent samples was fitted using the *lmFit()* function. Next, the *eBayes()* function is used to calculate the moderated t-statistics, moderated F-statistics and log-odds of differential expression by empirical Bayes moderation of the standard errors towards a common value. P values were adjusted using the Benjamini–Hochberg false discovery rate (FDR). Only DEGs with FDR-adjusted P values < 0.05 were considered for further analysis (**Additional file 3**).

Genes correlated with arthritis severity score ("Correlated genes"). For every cell type, we divided cells by mouse of origin and calculated the mean gene expression for every gene. Next, we correlated the mean gene expression of a cell type with the paired arthritis severity score using a Pearson correlation. To investigate whether the correlation was stronger than expected by chance, a bootstrap algorithm was employed that randomly deviated cell type and mouse-specific mean expression values by -0.2 to 0.2. P values for every gene were derived by comparison of the gene's correlation coefficient to the bootstrap mean correlation coefficient and standard deviation thereof. Only genes with $P < 0.05$ were considered for further analysis.

Largest connected component. The largest connected component (LCC) of a disease-associated gene set in a given PPIN was identified through application of the *induced_subgraph()* function of the igraph R package *(3)*. The largest such subgraph was selected for further analysis.

Pathway enrichment analysis using KEGG pathways. Human KEGG pathways were downloaded from KEGG (6) (January 2020) using the KEGGREST R package. A Fisher enrichment analysis was conducted to identify which pathways were significantly enriched with a specific gene set of interest. P values for all pathway enrichments were FDR adjusted. Pathway genes with $P < 0.05$ were considered gene sets of interest in further analysis.

Valdiation in the HuRI protein–protein interaction network. For validation, initial network outcomes were replicated using the smaller yet unbiased HuRI PPIN (8). Network proximity calculations were repeated in the same manner as previously described. Genes that were not included in the HuRI PPIN were removed from the list of drug targets as well as the gene sets of interest prior to calculation. As a result, the calculation included 888 drugs (compared to 1,840 in the literature-curated PPIN calculations), of which 30 were known RA drugs. Generally, recall and precision were slightly lower among the candidates in the HuRI-based network proximity screening (**Additional file 1: Fig. S20**) but were otherwise comparable to results in the literature-curated PPIN (**Additional file 1: Fig. S18 & S19**).

Pseudobulk RNA-seq data were used as input for Connectivity-Map. Since there are no alternative systematic methods that compute drug prioritization based on single-cell data, we compare our method with CMAP (13), which outputs a drug ranking by using signatures of bulk data and drug targets associated with these signatures. First, we converted healthy and sick scRNA-seq samples from AIA mice into pseudobulk expression by using *AggregateExpression*() in Seurat (14). Cells from each mouse were sampled twice to create two bulk profiles per mouse to be able to reach significance in later DEG calculations. Next, we computed DEGs between healthy and sick pseudobulk samples using the limma package as described above (12), which resulted in 1,105 and 23 genes that were up- and downregulated, respectively. The above approach ensured maximum comparability between methods, given that the transformation of scRNA-seq data to bulk data allows the same data set to be used for comparison of scDrugPrio and CMAP.

For its computations, the CMAP method needs a database containing information about genes that are targeted by each drug in the repository. To make a relevant comparison between CMAP and scDrugPrio, we used the 1840 DrugBank-derived drugs that entered network analysis in

scDrugPrio as such a database. We sorted significant DEGs based on absolute log-fold change before inputting the by CMAP required 150 top DEGs.

CMAP returned a ranked list including all 1840 drugs; however, only 95 drugs had a predicted CMAP effect > 0. Five approved RA drugs were found among these 95 drugs (**Additional file 1: Fig. S20)**. After the literature search had been performed (similar to the literature search performed for the AIA data), 20 additional drugs were suggested to have an effect, and 10 drugs had been studied previously and were either found to have no effect or exacerbating effects on disease. The precision for approved RA drugs was 5.3% (compared to 22% for scDrugPrio) and improved to 26.3% following the literature search (compared to 62% for scDrugPrio). Of the drugs that had been previously tested, 71.4% had shown success (compared to 95.4% for scDrugPrio).

Robustness analysis of scDrugPrio

To evaluate parameter dependency and thereby repeatability and reproducibility, we applied scDrugPrio to the AIA data set varying set cut-offs (**Additional file 1: Fig. S22**). Such cut-offs included 1) the number of DEGs used for network calculations, including network proximity and intracellular centrality calculations, 2) the significance of z_c (set to one-sided $P < 0.05$), 3) the network distance cut-off ($d_c < 1$), 4) pharmacological filtering criteria of a drug having to counteract the fold change of at least one targeted DEG, and 5) the background gene cut-off used during NicheNet ligand activity analysis for creation of the MCDM. The biopharmacological criteria (4) required manual evaluation, which complicates permutation. However, permutation was also deemed unnecessary, given that the biopharmacological criteria, applied to the same underlying data, would be expected to select/exclude the same set of drug candidates in every run.

To test the robustness of scDrugPrio to the other set cut-offs, we selected and ranked drug candidates over a variety of cut-offs. For the number of DEGs, no cut-off was originally set for the AIA data, resulting in up to 12,769 DEGs in the network calculations. Four more DEG cut-offs were chosen through random sampling within a preset range from 8,000 DEGs to the original cut-off. Following random sampling of the number of DEGs, network proximity calculations were performed using the original cut-off as well as 12,558, 12,148, 10,561 and 8,921 DEGs. The same

numbers of DEGs were used for intracellular centrality calculations. Drug selection was conducted using all possible combinations of $d_c < 0.8, 0.9, 1.0, 1.1$ or 1.2 and $P(z_c) < 0.01, 0.03, 0.05, 0.07, 0.09$, resulting in a total of 25 combinations of z_c-derived P values and d_c. The original background gene cut-off was $Ea(i) \geq 0.2$. We calculated NicheNet-derived intercellular centralities for a range of cut-offs spanning from 0.10 to 0.28 using 0.02 increments. Using all possible cut-off combinations for 1-3) and 5), we derived 1250 different sets of ranked drug candidates.

Aiming to evaluate robustness, the ranks of drug candidates were compared to the original drug ranking using Pearson correlation. A Pearson correlation coefficient of 1 would represent a perfect replication of the original drug ranking despite changed cut-offs. Our analysis showed a median [min – max] Pearson correlation coefficient of 0.861 [0.704 – 0.999], indicating that scDrugPrio was a stable and relocatable performance over the chosen threshold ranges (**Additional file 1: Fig. S22**). The number of DEGs seemed to affect the correlation coefficient the most, followed by the z_c cut-off. P values were Bonferroni corrected and remained significant in all instances (**Additional file 1: Fig. S22**).

Interindividual heterogeneity among sick patients/samples

To explore the variation between patients/samples, we compared 1) cell type proportions, 2) gene expression profiles and 3) examination of latent features of the non-batch corrected data. Interindividual differences in cell type proportions were explored by the application of the chi-square test to the proportions in patient samples. Heterogeneity between patients' gene expression profiles was explored through a random forest approach as described in the Methods section. Latent feature comparison was conducted visually through tSNE visualization of sick cell latent features derived from DCA (15). Cells were coloured based on the patient/sample to which they belonged. This allows for a visual appreciation of general molecular differences between patients. As seen, latent features for AIA mice show that mice mix well (**Additional file 1: Fig. S2c**), speaking to no underlying differences in their gene expression profiles. However, MS (**Additional file 1: Fig. S6a,b**) and CD patients (**Additional file 1: Fig. S9a,b**) show a more nonoverlapping pattern, indicating heterogeneity.

To understand whether heterogeneity between patient samples could be due to clinical factors, such as sex and disease progression, we downloaded available clinical data for the MS patients. We performed a correlation analysis between continuous clinical variables and cell type proportions of individual subjects (**Additional file 1: Fig. S22**). Analysing patient heterogeneity, we derived cell type proportions from the clustering of the non-batch-corrected MS data. The clinical variables included age as well as standard CSF parameters of all included patients, namely, total cells per µl, granulocytes per µl, red blood cells per µl, lymphocytes per µl, glucose (mg/d), lactate (mmol/l), and protein (mg/l). From these parameters, granulocytes and red blood cell counts were excluded because they did not show any meaningful variation between patients.

We tested the dependency between the cell type proportions of each of the IIH and MS subjects and their associated clinical data. We calculated Spearman correlations to identify if any of the cell type proportions significantly correlated with a continuous clinical variable (**Additional file 1: Fig. S22a**; **Additional file 4**). A significant correlation was found between:

1. Age and
    a. Activated terminally differentiated effector CD8+ T cells (P = 0.025, RHO = 0.64)
2. Total number of cells per ml and:
    a. Naïve CD4+ T cells (P = 0.04, RHO = -0.63),
    b. Early activated CD4+ memory T cells (P = 0.02, RHO = 0.69),
    c. Activated terminally differentiated effector CD8+ T cells (P = 0.001, RHO = -0.84)
3. Number of lymphocytes and:
    a. Naïve CD4+ T cells (P = 0.03, RHO = -0.71)
    b. Early activated CD4+ memory T cells (P = 0.02, RHO = 0.74)
    c. Activated terminally differentiated effector CD8+ T cells (P = 0, RHO = -0.93)
4. Lactate concentration in CSF in nmol/l and:
    a. Resting CD4+ memory T-cell 1 (P = 0.02, RHO = -0.72)
    b. Early activated CD8+ effector memory cells (P = 0.01, RHO = 0.75),
    c. Resting CD4+ memory T-cell 3 (P = 0.04, RHO = -0.66)
    d. Plasma cells (P = 0.01, RHO = 0.78).

Taken together, this strongly supported our cell typing approach, as the clinically measured lymphocyte count correlated well with the proportion of CD4+ and CD8+ typed cell clusters per patient. Interestingly, even age was correlated with cell type proportions of CD8+ T cells.

Furthermore, we acquired categorical clinical data in the form of the following variables:

- #stFLAIRles; number of supratentorial FLAIR hyperintense lesions in magnetic resonance imaging.
- MRI DIS+; criteria for dissemination in space fulfilled by MRI at the time of lumbar puncture. Gd+; any gadolinium-enhancing lesion detected on MR.
- OCB DIT+; criteria for dissemination in time fulfilled by the presence of oligoclonal bands in CSF at the time of lumbal puncture.
- Clinical DIT+; criteria for dissemination in time fulfilled by the presence of at least two clinical relapses.
- EDSS DC; Kurtzke Expanded Disability Status Scale (EDSS) score at discharge, i.e., after treatment of the relapse occurring at the lumbar puncture.
- EDSS FU; latest available clinical follow-up in months after lumbar puncture.
- OCB+; any oligoclonal band (OCB) detected in CSF.
- OCB type and OCB were classified as being either undetectable, restricted to CSF, detected in serum and additionally in CSF, or not determined.
- CSF index; CSF/serum indices for albumin and immunoglobulin G (IgG) were calculated. The CSF index was evaluated as being either unaffected (none), showing intrathecal IgG synthesis (Ig only), showing barrier dysfunction (barrier only), or showing both intrathecal IgG synthesis and barrier dysfunction (barrier and Ig).
- Sex (male or female).
- Prior relapse; number of clinical relapses prior to the one occurring at lumbar puncture.

For the purpose of finding associations between categorical clinical variables and cell type proportions, we implemented a logistic regression (glm() R function, package stats v.4.0.4). Cell type proportions were used as predictors and individual clinical data as the predicted variable (for example, to test if the cell type proportions are associated with sex, we created a model where cell type proportions were independent variables and sex is the predicted variable). We found no

significant association between any of the independent variables and categorical clinical variables (**Additional file 4**).

3D-network figure for drug candidate interactions with plasma cell DEGs of AIA mice

To understand the interactions between DEGs and drug candidates, selected based on $z_c < -1.64$ and $d_c < 1$, we created a 3D visualisation (https://scpred.shinyapps.io/3D_network/) for the most central cell type, activated B cells, in the AIA mouse data. Interactions between DEGs (blue) represent protein–protein interactions (PPIs) described in the literature-curated PPI network by do Valle et al. (16). DEG node size is based on fold change. Drug candidates are connected to their respective gene drug targets by edges. Potential drug candidates are shown in red. Established drugs for human rheumatoid arthritis are represented in yellow. The higher the absolute value of a drug on the Y-axis, the higher the drug rank. Drug candidates that counteracted at least one DEG fold change received positive Y-axis values, while drug candidates that did not counteract the fold change of any targeted DEG received negative Y-axis values. By clicking on one of the nodes, the neighbouring nodes are highlighted. Details on the computational environment are provided in https://github.com/SDTC-CPMed/scDrugPrio.

Supplementary Tables

Table S1. Library preparation and sample pooling.			
**Sample**	n libraries prepared	n samples sequenced and merged	n samples per Illumina array
Joint_Healthy_mouse_1	4	4	3
Joint_Healthy_mouse_2	4	4	3
Joint_Healthy_mouse_3	4	5	3 (1 for one of the libraries)
Joint_Healthy_mouse_4	4	4	3
Joint_Sick_mouse_1	4	4	3
Joint_Sick_mouse_3	4	4	3
Joint_Sick_mouse_4	4	4	3
Joint_Sick_mouse_5	4	4	3
Joint_Sick_mouse_6	4	4	3

To increase the read depth for each sample, multiple libraries were prepared and sequenced. For healthy mouse 3, one of the libraries was sequenced twice, wherein one was sequenced alone on one array, without pooling with other samples.

**Table S2. Drug concentrations for *in vitro* validation studies.**	
**Drug**	**In vitro concentrations (low, medium, high)**
**Auranofin**(17, 18)	10 nM, 50 nM and 250 nM
**Dimethyl fumarate(19)**	5 µM, 25 µM and 100 µM
**Irbesartan(20)**	2 µM, 10 µM and 50 µM
**Amrinone(21)**	10 µM, 50 µM and 250 µM
**Isosorbide(22, 23)**	5 µM, 25 µM and 100 µM
**Adapalene(24, 25)**	200 nM,1 µM and 5 µM

References:

1. Gawel DR, Serra-Musach J, Lilja S, Aagesen J, Arenas A, Asking B, et al. A validated single-cell-based strategy to identify diagnostic and therapeutic targets in complex diseases. Genome Med. 2019;11(1):47.
2. Buniello A, MacArthur JAL, Cerezo M, Harris LW, Hayhurst J, Malangone C, et al. The NHGRI-EBI GWAS Catalog of published genome-wide association studies, targeted arrays and summary statistics 2019. Nucleic Acids Res. 2019;47(D1):D1005-d12.
3. Csardi G, Nepusz T. The igraph software package for complex network research. InterJournal, complex systems. 2006;1695(5):1-9.
4. Martin JC, Chang C, Boschetti G, Ungaro R, Giri M, Grout JA, et al. Single-Cell Analysis of Crohn's Disease Lesions Identifies a Pathogenic Cellular Module Associated with Resistance to Anti-TNF Therapy. Cell. 2019;178(6):1493-508.e20.
5. Hamosh A, Scott AF, Amberger J, Valle D, McKusick VA. Online Mendelian Inheritance in Man (OMIM). Hum Mutat. 2000;15(1):57-61.
6. Kanehisa M. Toward understanding the origin and evolution of cellular organisms. Protein Sci. 2019;28(11):1947-51.
7. Guney E, Menche J, Vidal M, Barabasi AL. Network-based in silico drug efficacy screening. Nat Commun. 2016;7:10331.
8. Luck K, Kim D-K, Lambourne L, Spirohn K, Begg BE, Bian W, et al. A reference map of the human binary protein interactome. Nature. 2020;580(7803):402-8.
9. Woetzel D, Huber R, Kupfer P, Pohlers D, Pfaff M, Driesch D, et al. Identification of rheumatoid arthritis and osteoarthritis patients by transcriptome-based rule set generation. Arthritis Res Ther. 2014;16(2):R84.
10. Tasaki S, Suzuki K, Kassai Y, Takeshita M, Murota A, Kondo Y, et al. Multi-omics monitoring of drug response in rheumatoid arthritis in pursuit of molecular remission. Nature communications. 2018;9(1):2755-.
11. Davis S, Meltzer PS. GEOquery: a bridge between the Gene Expression Omnibus (GEO) and BioConductor. Bioinformatics. 2007;23(14):1846-7.
12. Ritchie ME, Phipson B, Wu D, Hu Y, Law CW, Shi W, et al. limma powers differential expression analyses for RNA-sequencing and microarray studies. Nucleic Acids Res. 2015;43(7):e47.
13. Lamb J, Crawford ED, Peck D, Modell JW, Blat IC, Wrobel MJ, et al. The Connectivity Map: using gene-expression signatures to connect small molecules, genes, and disease. Science. 2006;313(5795):1929-35.
14. Butler A, Hoffman P, Smibert P, Papalexi E, Satija R. Integrating single-cell transcriptomic data across different conditions, technologies, and species. Nature Biotechnology. 2018;36(5):411-20.
15. Eraslan G, Simon LM, Mircea M, Mueller NS, Theis FJ. Single-cell RNA-seq denoising using a deep count autoencoder. Nature Communications. 2019;10(1):390.

16. do Valle IF, Roweth HG, Malloy MW, Moco S, Barron D, Battinelli E, et al. Network medicine framework shows that proximity of polyphenol targets and disease proteins predicts therapeutic effects of polyphenols. Nature Food. 2021;2(3):143-55.
17. Park SH, Lee JH, Berek JS, Hu MC. Auranofin displays anticancer activity against ovarian cancer cells through FOXO3 activation independent of p53. Int J Oncol. 2014;45(4):1691-8.
18. Zhang H, Rose BJ, Pyuen AA, Thamm DH. In vitro antineoplastic effects of auranofin in canine lymphoma cells. BMC Cancer. 2018;18(1):522.
19. Diebold M, Sievers C, Bantug G, Sanderson N, Kappos L, Kuhle J, et al. Dimethyl fumarate influences innate and adaptive immunity in multiple sclerosis. J Autoimmun. 2018;86:39-50.
20. Zhao Y, Watanabe A, Zhao S, Kobayashi T, Fukao K, Tanaka Y, et al. Suppressive effects of irbesartan on inflammation and apoptosis in atherosclerotic plaques of apoE-/- mice: molecular imaging with 14C-FDG and 99mTc-annexin A5. PLoS One. 2014;9(2):e89338.
21. Chanani NK, Cowan DB, Takeuchi K, Poutias DN, Garcia LM, Nido PJd, et al. Differential Effects of Amrinone and Milrinone Upon Myocardial Inflammatory Signaling. Circulation. 2002;106(12_suppl_1):I-284-I-9.
22. Rolland PH, Berenger FP, Cano JP. In vitro evidence of an endothelial cell-dependent antiplatelet activity for isosorbide dinitrate, but not for its 2- and 5-mononitrate metabolites. J Pharmacol Exp Ther. 1987;240(1):234-40.
23. Trongvanichnam K, Mitsui-Saito M, Ozaki H, Karaki H. Effects of chronic oral administration of isosorbide dinitrate on in vitro contractility of rat arterial smooth muscle. Jpn J Pharmacol. 1996;71(2):167-73.
24. Tenaud I, Khammari A, Dreno B. In vitro modulation of TLR-2, CD1d and IL-10 by adapalene on normal human skin and acne inflammatory lesions. Exp Dermatol. 2007;16(6):500-6.
25. Zuliani T, Khammari A, Chaussy H, Knol AC, Dréno B. Ex vivo demonstration of a synergistic effect of Adapalene and benzoyl peroxide on inflammatory acne lesions. Exp Dermatol. 2011;20(10):850-3.
